# Charge-switchable zwitterionic polycarboxybetaine particle as an intestinal permeation enhancer for efficient oral insulin delivery

**DOI:** 10.7150/thno.54176

**Published:** 2021-03-04

**Authors:** Yan Li, Weihong Ji, Huan Peng, Ruichen Zhao, Tianlu Zhang, Zhiguo Lu, Jun Yang, Ruiyuan Liu, Xin Zhang

**Affiliations:** 1 State Key Laboratory of Biochemical Engineering, Institute of Process Engineering, Chinese Academy of Sciences, Beijing 100190, PR China.; 2 University of Chinese Academy of Sciences, Beijing 100049, PR China.

**Keywords:** oral administration, charge-switchable, polycarboxybetaine, intestinal permeation enhancer, insulin

## Abstract

Insulin, a peptide hormone, is one of the most common and effective antidiabetic drugs. Although oral administration is considered to be the most convenient and safe choice for patients, the oral bioavailability of insulin is very low due to the poor oral absorption into blood circulation. Intestinal epithelium is a major barrier for the oral absorption of insulin. Therefore, it is vital to develop intestinal permeation enhancer to increase the antidiabetic efficacy of insulin after oral administration.

**Methods:** Charge-switchable zwitterionic polycarboxybetaine (PCB) was used to load insulin to form PCB/insulin (PCB/INS) particles through the electrostatic interaction between positively charged PCB in pH 5.0 and negatively charged insulin in 0.01 M NaOH. The opening effect of PCB/INS particles on intestinal epithelium was evaluated by detecting the changes of claudin-4 (CLDN4) protein and transepithelial electrical resistance (TEER) after incubation or removal. The mechanism was further elucidated based on the results of Western blot and fluorescence images. The PCB/INS particles were then used for type 1 diabetes mellitus therapy after oral administration.

**Results:** PCB could load insulin with the loading efficiency above 86% at weight ratio of 8:1. PCB/INS particles achieved sustained release of insulin at pH 7.4 due to their charge-switchable ability. Surprisingly, PCB/INS particles induced the open of the tight junctions of intestinal epithelium in endocytosis-mediated lysosomal degradation pathway, which resulted in increased intestinal permeability of insulin. Additionally, the opening effect of PCB/INS particles was reversible, and the decreased expression of CLDN4 protein and TEER values were gradually recovered after particles removal. In streptozotocin-induced type 1 diabetic rats, oral administration of PCB/INS particles with diameter sub-200 nm, especially in capsules, significantly enhanced the bioavailability of insulin and achieved longer duration of hypoglycemic effect than the subcutaneously injected insulin. Importantly, there was no endotoxin and pathological change during treatment, indicating that PCB/INS particles were safe enough for *in vivo* application.

**Conclusion:** These findings indicate that this system can provide a platform for oral insulin and other protein drugs delivery.

## Introduction

Diabetes mellitus is a chronic disease characterized by elevated blood glucose, which currently affects more than 450 million people worldwide 1. Insulin, a peptide hormone, is one of the most common and effective antidiabetic drugs. Insulin can promote the absorption of glucose and its conversion into glycogen to reduce blood glucose level 2-4. Nowadays, insulin is used with multiple daily subcutaneous injections in clinic 5-7. However, the subcutaneous injection results in low patient compliance and safety issues 8,9. In comparison, oral administration is considered to be the most convenient and safe choice for patients because of high patient compliance and repeatable administration 10. Additionally, the oral insulin formulation can provide a better glucose homeostasis in the body as it more closely mimics the endogenous insulin pathway 11,12. Nonetheless, the oral bioavailability of insulin is very low due to the poor oral absorption into blood circulation.

Intestinal epithelium is a major barrier to the blood absorption of insulin after oral administration 13,14. The transport of hydrophilic macromolecules through intestinal epithelium relies on the paracellular route 15. However, the paracellular route is severely restricted by the presence of the tight junctions that are located at the luminal aspect of adjacent epithelial cells 16-18. Only molecules less than 1 nm in hydrodynamic radius are permitted to cross the tight junctions, and insulin cannot diffuse across due to its high molecular weight 19. The degree of sealing for the tight junctions is highly dynamic, which depends on the extent of external stimuli 20. Therefore, it is possible to develop efficient intestinal permeation enhancers that can enhance the permeability of insulin across the intestinal epithelium.

Several intestinal permeation enhancers have been developed to aid the transport of protein drugs across the intestine. The mechanisms are thought to open the tight junctions between the intestinal epithelial cells or facilitate transcellular transport through an increase in plasma membrane permeability 21,22. Although some of these intestinal permeation enhancers have been evaluated in early-stage clinical trials, a relatively poor translation has been achieved until now 23. The major reasons are that only a small portion of these intestinal permeation enhancers can be used into solid-dose formulations and some have high cytotoxicity 24,25. It is recognized that an ideal intestinal permeation enhancer should be nontoxic, and have strong dosage form assembling ability for protein drug delivery.

Zwitterionic polycarboxybetaine (PCB) has cationic quaternary ammonium group and anionic carboxyl acid group alone, which is neutral under physiological environment with pH 7.4. In our previous studies, it has been demonstrated that the protonation of carboxyl acid group in acidic environment could transfer PCB from neutral to positively charged 26-28. Insulin is negatively charged when the pH value is above its isoelectric point (pH 5.3) 29,30. Therefore, PCB has the potential of loading insulin via electrostatic interaction. In this study, we described the serendipitous discovery that charge-switchable zwitterionic PCB could complex insulin into dosage form and the PCB/insulin (PCB/INS) particles could open the tight junctions as an intestinal permeation enhancer. The study offered a promising candidate for oral protein drugs administration for diseases treatment.

## Results and Discussion

### PCB/INS particles with varied morphologies and sizes

Herein, PCB was synthesized via the reversible addition-fragmentation chain transfer polymerization (**Figure S1**). The chemical structure of PCB was characterized by ^1^H NMR (**Figure S2**), which indicated its successful synthesis. The degree of polymerization (DP) was further determined by gel permeation chromatography. PCB with molecular weight of 5,394, 8,497 and 28,180 Da were synthesized (**Figure S3**), and their corresponding DP was approximately 23, 36 and 122, respectively.

**Figure 1:**
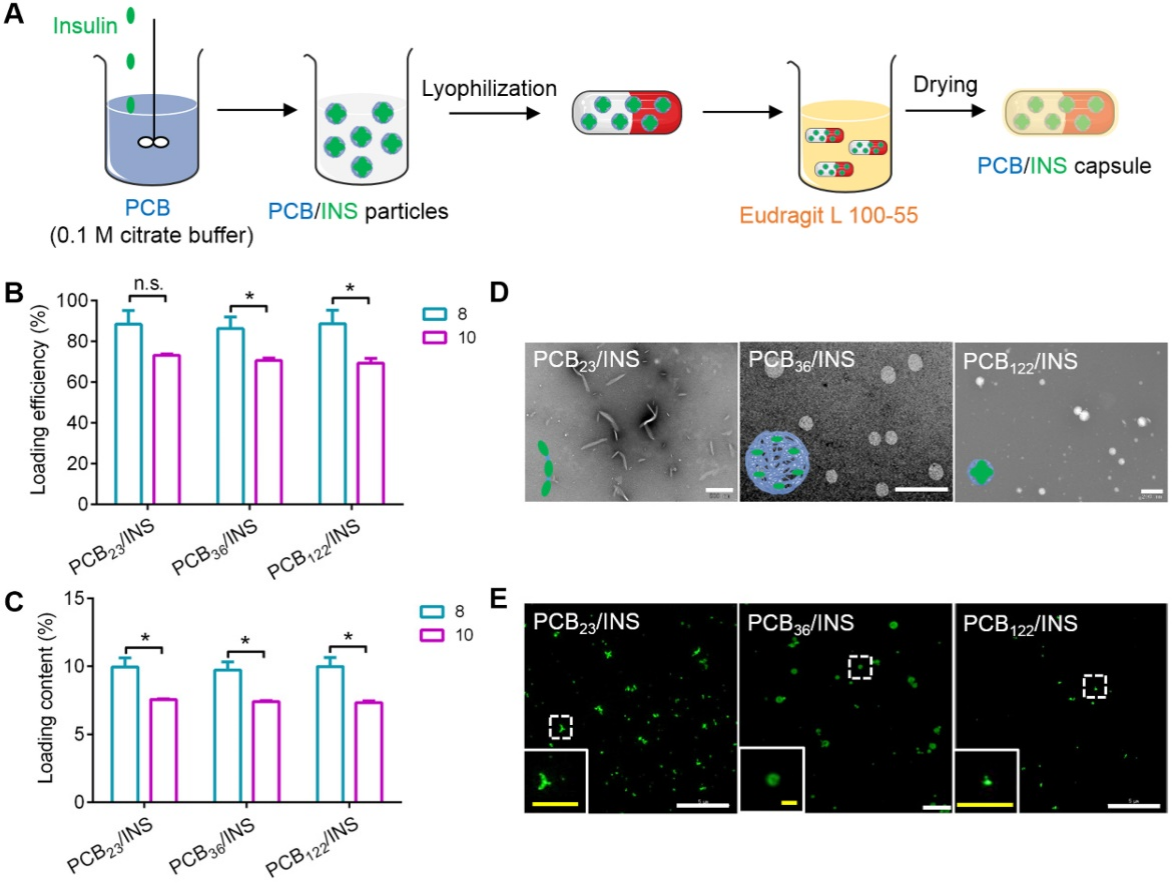
** Design and characterization of PCB/INS particles. (A)** The strategy for construction of PCB/INS particles/capsule. **(B)** The loading efficiency and **(C)** loading content of insulin for different PCB/INS particles at mass ratio of 8:1 and 10:1 between PCB and insulin, respectively.** (D)** TEM images of particles. The particles were negatively stained with phosphotungstic acid. The scale bar was 500 nm, 2 µm and 200 nm for PCB_23_/INS, PCB_36_/INS and PCB_122_/INS particles image, respectively. **(E)** The 3D-SIM images of the PCB_23_/INS, PCB_36_/INS and PCB_122_/INS particles with FITC-labeled insulin. The white scale bar: 5 µm. The yellow scale bar in insert images: 1 µm. The mean ± SD was shown (n = 3). n.s. > 0.05, *P < 0.05.

Next, PCB/INS particles were prepared through the electrostatic interaction between positively charged PCB in pH 5.0 and negatively charged insulin in 0.01 M NaOH at different mass ratios (**Figure 1A**). The diameters of particles were reduced with the increase of mass ratio between PCB and insulin (**Figure S4**). The diameters were stable at the mass ratio of 8:1. Furthermore, the loading efficiency and loading content of insulin were measured at the mass ratio of 8:1 and 10:1, respectively. As shown in **Figure 1B** and **1C**, the loading efficiency and loading content of insulin for particles at the mass ratio of 8:1 were higher than that at 10:1. Excess materials might impose an increased burden on patients to excrete. Therefore, particles with the mass ratio of 8:1 were selected and utilized for the following experiments. Additionally, the DP had almost no influence on the insulin encapsulation efficiency, and all particles exhibited above 86% at the mass ratio of 8:1 (**Figure 1B**).The transmission electron microscopy (TEM) images showed that PCB with different DP could form particles with different shapes (**Figure 1D**). PCB_23_/INS particles were linear with a length of approximately 500 nm. PCB_36_ loaded insulin forming spherical particles with a diameter of about 500 nm. PCB_122_/INS particles were spherical with a diameter of about 80 nm. Furthermore, the distribution of insulin in particles was observed with DeltaVision OMX V3 imaging system. The 3D-SIM images of particles confirmed that PCB with different DP could load insulin to form particles with different shapes (**Figure 1E**). This might be attributed to the different curvature of these polymers. PCB was prone to bend to form spherical structure with high DP. PCB/INS particles referred to these three formulations in the following studies.

### PCB/INS particles achieved controlled release of insulin

**Figure 2:**
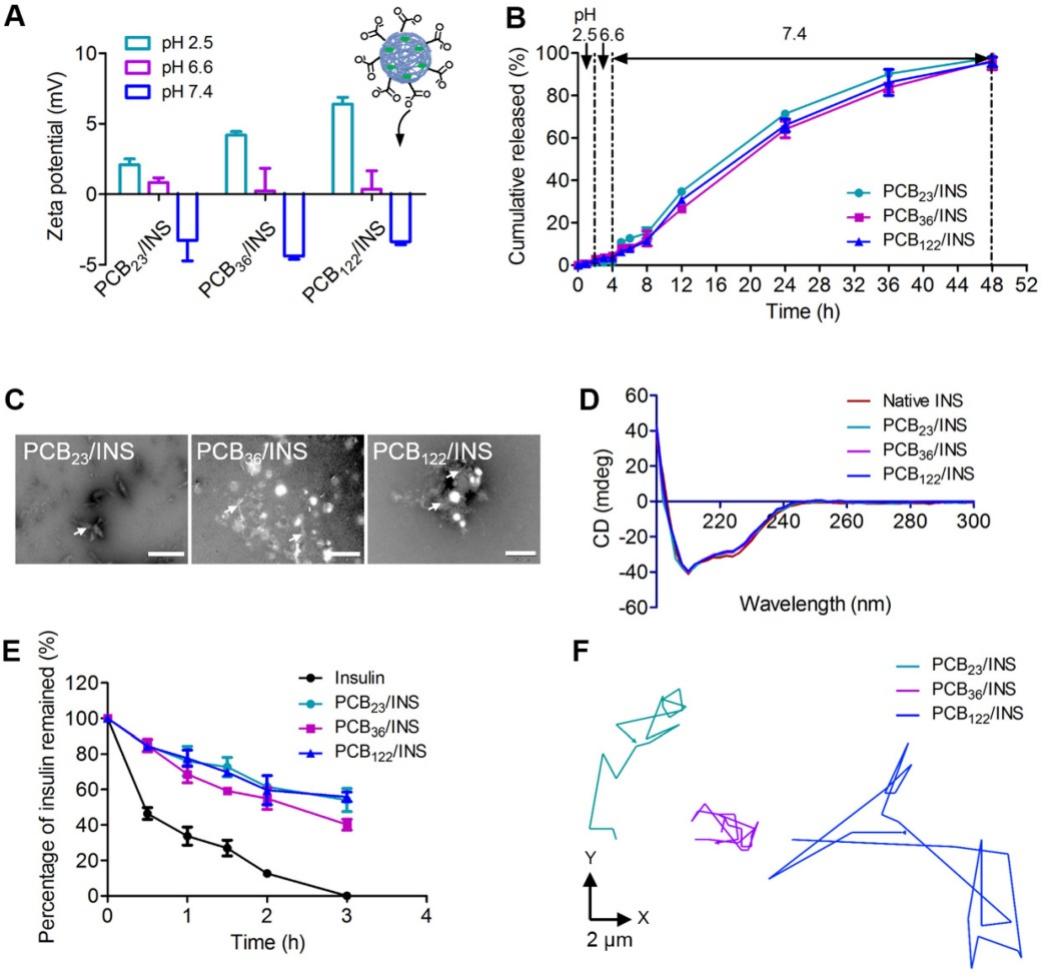
**The release and activity of insulin. (A)** The zeta potential of particles at different pH microenvironments. **(B)** Release profiles of insulin from particles at distinct pH microenvironment. **(C)** TEM images of PCB/INS particles in phosphate buffer saline (PBS) (pH 7.4) after 24 h incubation. The particles were negatively stained with phosphotungstic acid. The white arrows indicated the fragments. The scale bar was 500 nm, 1 µm and 200 nm for PCB_23_/INS, PCB_36_/INS and PCB_122_/INS particles image, respectively. **(D)** CD spectra of the native insulin and insulin released from the particles. **(E)** The percentage of insulin undergraded at different time points in the trypsin solution. **(F)** Representative motion trajectories of the PCB_23_/INS, PCB_36_/INS and PCB_122_/INS particles with FITC-labeled insulin in mucus at a time lapse of 7 s. The mean ± SD was shown (n = 3).

The pH environment of the gastrointestinal tract varies from the stomach to the small intestine. The pH 2.5, 6.6 and 7.4 were used to simulate the pH microenvironment in the stomach, duodenum, and the body fluid at the intercellular spaces between epithelial cells, respectively 31. The protonation of PCB at pH 2.5 increased the surface positively charge of particles (**Figure 2A**), which could result in stronger electrostatic adsorption between PCB and insulin. The particles diameters were stable (**Figure S5**) and the *in vitro* release profiles showed that there was almost no insulin released at pH 2.5 after 2 h incubation (**Figure 2B**). The zeta potential of all particles was decreased with the increase of pH value, which was attributed to the deprotonation of carboxyl acid groups (**Figure 2A**). A partial negative surface charge on the particles was generated, and the particles were changed to be anionic at pH 7.4. The diameters increased obviously at pH 7.4 due to the electrostatic repulsion between insulin and deprotonated carboxyl acid group after 2 h incubation (**Figure S5**). The anionic particles gradually started to sustained release of insulin. The cumulative released percentage was 95.9±2.2% for PCB_122_/INS particles at pH 7.4 after 48 h incubation. To further confirm this, the morphology of PCB/INS particles was observed by TEM after 24 h incubation at pH 7.4. Part of the particles were unstable and subsequently broke apart (**Figure 2C**). These results indicated that PCB/INS particles could achieve controlled release of insulin in the condition of intercellular spaces between epithelial cells.

The released insulin was collected and the structural change was investigated using the circular dichroism (CD) spectra. The CD spectrum of native insulin had two ellipticity angles at 208 and 222 nm that belonged to the α-helix structure of insulin 32,33. The CD spectra of insulin released from all particles were similar to that of native insulin (**Figure 2D**), which demonstrated that the formulation procedures had almost no influence on the conformation and activity of insulin. The ability of particles to defend insulin against trypsin was further evaluated. The insulin remained in particles was approximately 60% at 2 h (**Figure 2E**), which was about 4.7-fold to that of free insulin. All insulin was undergraded for free insulin in the trypsin solution at 3 h. These results suggested that the particles could protect the insulin against trypsin-degradation.

Intestinal mucus is a layer of slippery secretion, that covers and protects the underlying epithelium 34,35. The mucus can rapidly trap the foreign particles. The movement behavior of particles in mucus greatly influences the interaction of particles with epithelium and the delivery of insulin 36. The movement of PCB_23_/INS, PCB_36_/INS and PCB_122_/INS particles in mucus was recorded with confocal laser scanning microscope (CLSM), and analyzed by Volocity. As shown in **Figure 2F** and **Figure S6**, the PCB_122_/INS particles with smaller diameter spanned much longer distance than PCB_23_/INS and PCB_36_/INS particles at a time scale of 7 s. The faster diffusion was beneficial for the oral delivery of insulin across the mucus.

### PCB/INS particles enhanced the intestinal permeability of insulin in a manner of paracellular pathway

**Figure 3:**
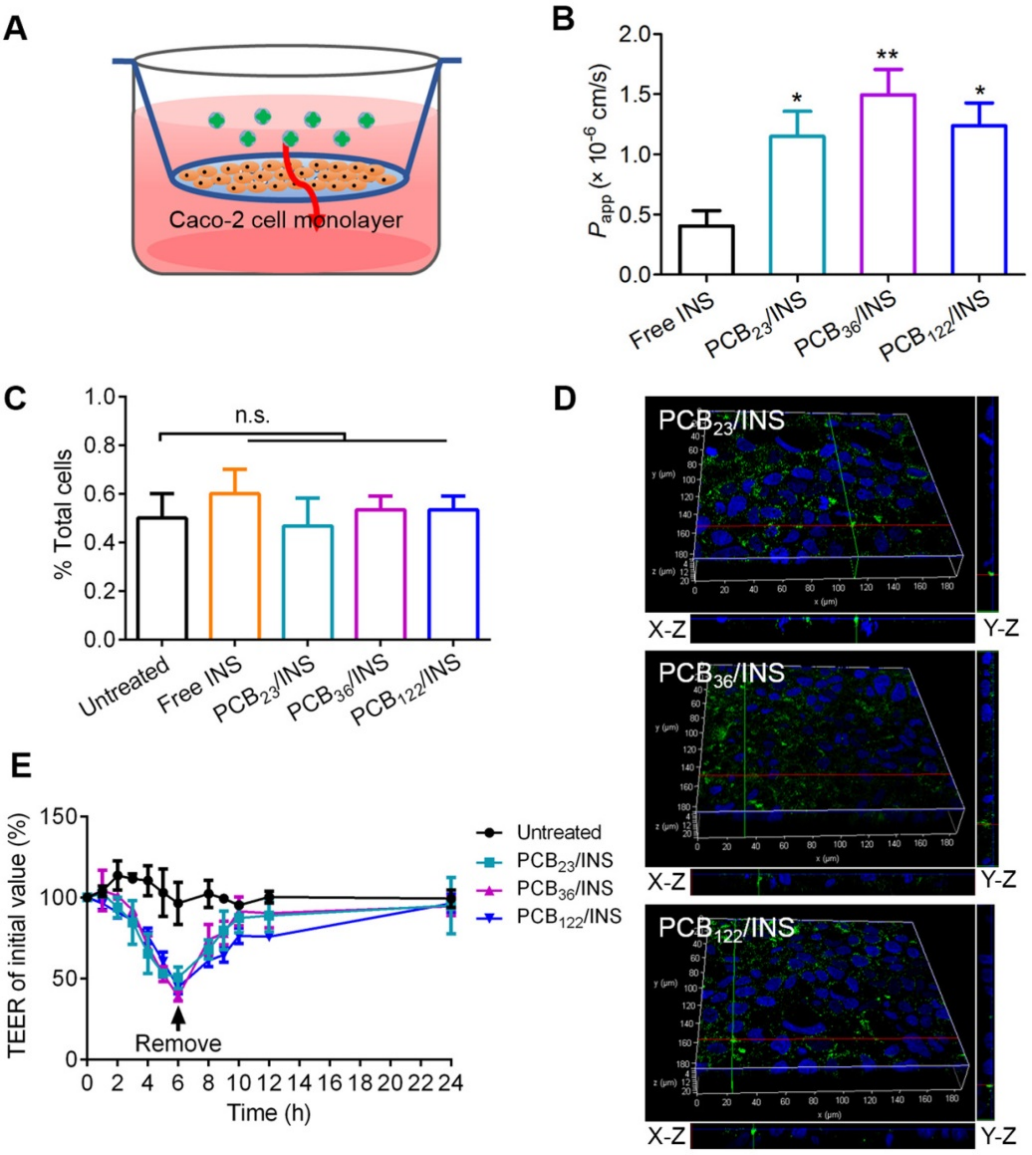
***In vitro* evaluation of the intestinal permeability and route of insulin across the intestinal epithelium from PCB/INS particles.** (**A**) The transwell^®^ model of the Caco-2 cell monolayer. (**B**) *P*_app_ values of free insulin and insulin from PCB/INS particles after incubating with Caco-2 cell monolayers in transwell^®^ for 24 h. (**C**) Cellular uptake of particles with FITC-labeled insulin after incubation with Caco-2 cells for 5 h detected by flow cytometry. (**D**) Three-dimensional fluorescence images of Caco-2 cell monolayers after incubation with the particles for 5 h. X-Z and Y-Z planes: the vertical planes. (**E**) Effect of PCB/INS particles on the TEER values of Caco-2 cell monolayers. Particles at a concentration of 5 µg PCB/mL were in Hanks' balanced salt solution (HBSS) buffer with pH 7.4. Particles were removed at 6 h. The monolayers were washed three times with HBSS buffer. The mean ± SD was shown (n = 3). *P < 0.05, **P < 0.01 in (B) with respect to free insulin. n.s. > 0.05.

Caco-2 cells, derived from human epithelial colorectal adenocarcinoma, have been widely accepted as an *in vitro* model of small intestine 37. 3-(4,5-Dimethylthiazol-2-yl)-2,5-diphenyltetrazolium bromide (MTT) assay showed that free insulin had a growth promoting effect on Caco-2 cells at the testing concentration (**Figure S7A**). The cell viability was slightly decreased with the increase of polymers concentration, and it was above 84.8% at 20 μg/mL PCB (**Figure S7B**). The biocompatibility of PCB/INS particles on Caco-2 cells was further studied using Alamar blue assay and MTT assay. The PCB/INS particles treatment had almost no negative effect on the cell proliferation and viability (**Figure S8**). The cell viability was approximately 90% even at 20 μg/mL PCB (**Figure S8B**), rendering these particles were suitable for *in vivo* application. Caco-2 cell monolayers in transwell^®^ were used to evaluate the transport ability of PCB/INS particles across the intestinal epithelium (**Figure 3A**). The apparent permeability coefficient (*P*_app_) was measured, which was defined as the rate of the appearance of the test insulin in the receiving compartment 38. The *P*_app_ values of insulin for three PCB/INS particles were significantly higher than that of free insulin (**Figure 3B**). The high *P*_app_ values indicated that PCB/INS particles could enhance the transepithelial transport efficiency of insulin.

To detect how the insulin from PCB/INS particles across the Caco-2 cell monolayer, the fluorescence signal of FITC-labeled insulin was detected in cells by flow cytometry. After washing with PBS and digesting with trypsin, no green fluorescence signal was detected in single cell with 5 h incubation (**Figure 3C**). The data meant that FITC-labeled insulin from PCB/INS particles did not utilize the transcellular route to cross the intestinal cells. Additionally, three-dimensional visualization of cells demonstrated that the green fluorescence signals were from top to bottom of the cell monolayers after particles treatment for 5 h (**Figure 3D**). Therefore, the insulin from PCB/INS particles crossed the intestinal cells in a manner of paracellular pathway.

Furthermore, the transepithelial electrical resistance (TEER) was measured, which could reflect the ion permeability of the monolayer. As the tight junctions open, the TEER would be reduced due to the ion passages through the paracellular route 10. There was a progressive decrease of TEER with PCB/INS particles incubation (**Figure 3E**). After particles removal, the TEER values were gradually recovered. Therefore, the increased intestinal permeability of insulin was attributed to the intestinal permeation enhancer effect of the PCB/INS particles by opening the tight junctions between the intestinal epithelial cells, and the action was reversible after the particles removal 14,17.

### PCB/INS particles induced reversible redistribution of the tight junction-associated proteins

**Figure 4:**
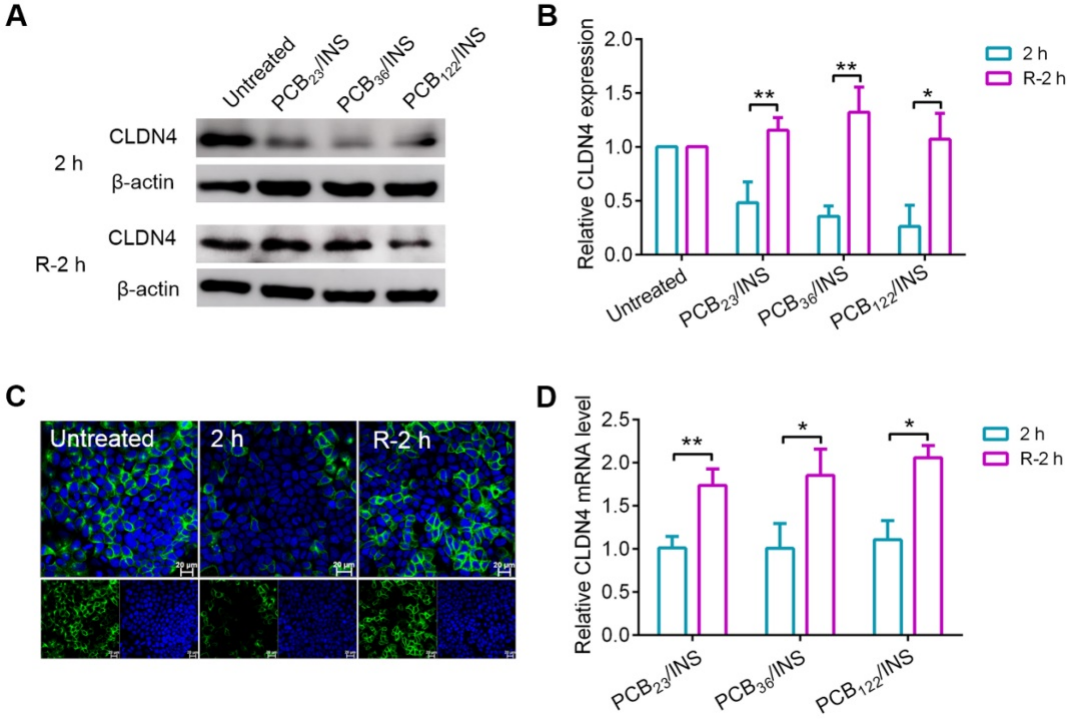
**The CLDN4 protein and mRNA level of Caco-2 cell monolayers after treatment. (A)** Western blot results of CLDN4 after 2 h of PCB_23_/INS, PCB_36_/INS and PCB_122_/INS particles incubation at a concentration of 5 µg PCB/mL or removing.** (B)** Western bands were scanned and normalized to the gray values of internal control β-actin. Quantified results of CLDN4 in (A) using ImageJ. The data was relative to the untreated control in each Western band. The untreated control was settled to 1. **(C)** Fluorescence images collected by CLSM after 2 h of PCB_122_/INS particles treatment or removal. CLDN4 was shown in green, and cell nuclei stained with DAPI were in blue. **(D)** Relative CLDN4 mRNA level in Caco-2 cells after 2 h of PCB_23_/INS, PCB_36_/INS and PCB_122_/INS particles incubation or removal. The mean ± SD was shown (n = 3). *P < 0.05, **P < 0.01.

The tight junctions are composed of a complex combination of transmembrane integral proteins that form a network between adjacent cell membranes. Their distribution and expression levels play a critical role in regulating the tight junction barrier of paracellular route 39. In order to assess the effect of PCB/INS particles on the level of transmembrane integral proteins, the expression level of claudin-4 (CLDN4), one of the transmembrane integral proteins in intestinal tissue, was examined via Western blot 40,41. Cells were exposed to PCB/INS particles for 2 h, and particles were then removed with PBS washing. The level of CLDN4 protein was down-regulated with the incubation of PCB/INS particles at a concentration of 5 μg PCB/mL regardless of its DP (**Figure 4A and 4B**). The expression was recovered after particles removal. Additionally, the concentration had an influence on the down-regulation effect of PCB/INS particles. PCB/INS particles at a concentration of 5 μg PCB/mL resulted in a significant lower CLDN4 protein level than the particles at a concentration of 1 μg PCB/mL (**Figure S9**). The immunofluorescence staining showed that CLDN4 was concentrated at the cell-cell contact and reduced after PCB/INS particles treatment (**Figure 4C and Figure S10**). Therefore, PCB/INS particles opened the tight junctions between Caco-2 cells by reducing the transmembrane integral proteins. Importantly, this effect was reversible without permanently destroying the cell monolayers. This is vital as the tight junctions are also biological barrier for restricting pathogen access 42,43.

It was noted that there was no apparent change in CLDN4 mRNA level 2 h after PCB/INS particles treatment (**Figure 4D**). The level of CLDN4 mRNA was increased 2 h after PCB/INS particles removal. The high expression of *Cldn4* resulted in the eventually back of CLDN4 protein to the normal level. Therefore, the changes of CLDN4 protein might be attributed to the redistribution of protein.

### The mechanism for PCB/INS particles opening the tight junctions

**Figure 5:**
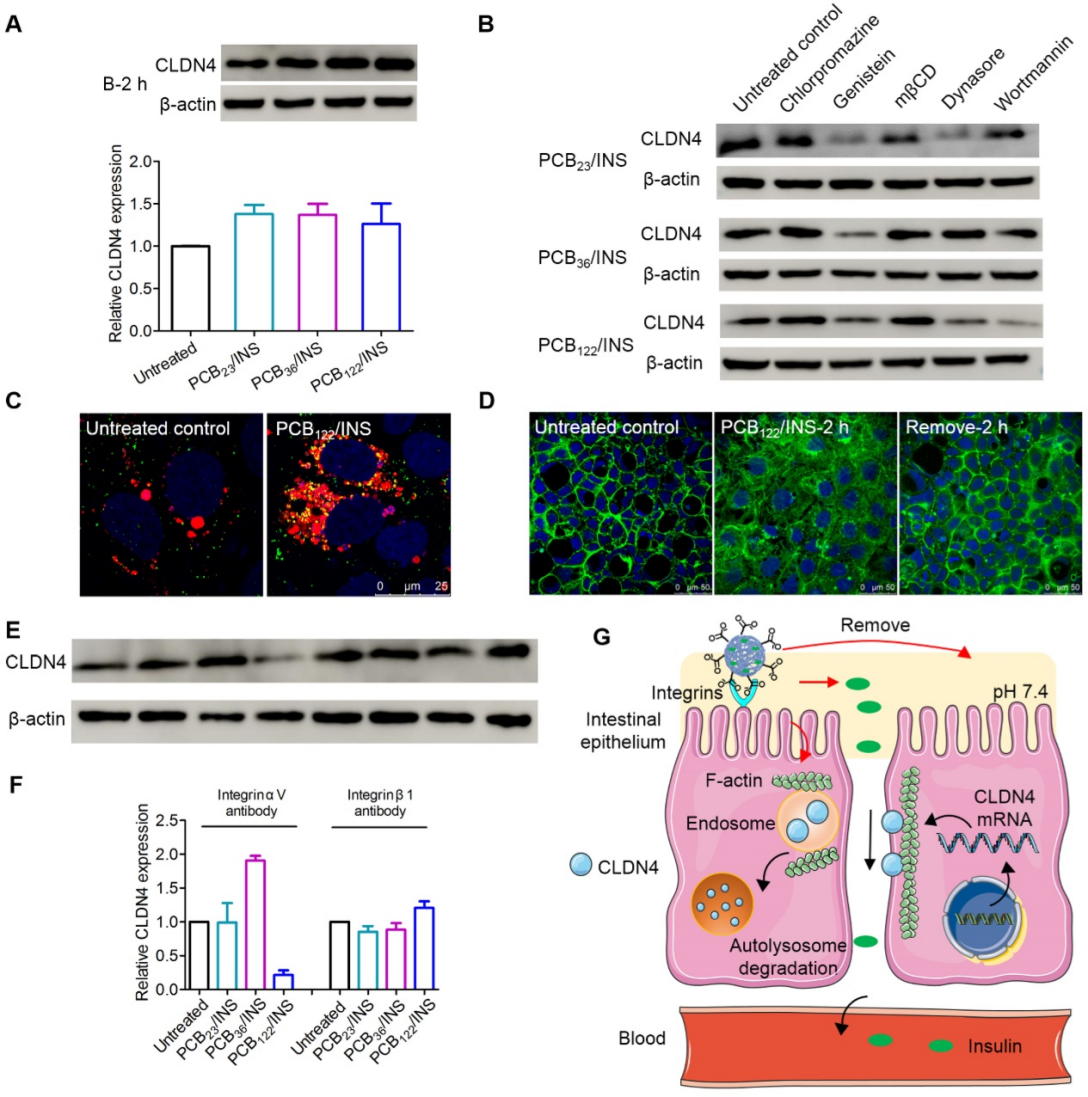
**The mechanism for PCB/INS particles opening the tight junctions. (A)** Western blot results of CLDN4 expression after 2 h of PCB_23_/INS, PCB_36_/INS and PCB_122_/INS particles treatment in the presence of bafilomycin A1. Western bands were scanned and normalized to the gray values of internal control β-actin. The results were quantified using ImageJ. **(B)** Western blot results of CLDN4 expression after 2 h of PCB_23_/INS, PCB_36_/INS and PCB_122_/INS particles treatment in the presence of various endocytic inhibitors. **(C)** Fluorescence images of Caco-2 cell monolayers after incubation with PCB_122_/INS particles for 2 h. CLDN4 was shown in green. Endosomes/lysosomes stained with LysoTracker deep red were in red. Cell nuclei stained with DAPI were in blue. **(D)** Fluorescence images collected by CLSM. F-actin stained with FITC-labeled phalloidin was shown in green, and the cell nuclei stained with DAPI were in blue. **(E)** Western blot results of CLDN4 expression after 2 h of PCB_23_/INS, PCB_36_/INS and PCB_122_/INS particles treatment in the presence of integrin αV antibody or integrin β1 antibody. **(F)** Western bands were scanned and normalized to the gray values of internal control β-actin. Quantified results of CLDN4 in (E) using ImageJ.** (G)** The schematic diagram for the mechanism. For Western blot, the data was relative to the untreated control in each Western band. The untreated control was settled to 1. The mean ± SD was shown (n = 3).

Bafilomycin A1 is a proton pump inhibitor, which selectively inhibits the vacuolar H^+^-ATPase and prevents the acidification of lysosomes 44. The bafilomycin A1 treatment could prevent the degradation of CLDN4 protein (**Figure 5A**). This data suggested that the degradation of CLDN4 occurred most likely via the lysosomal degradation pathway. We further probed the mechanism underlying the cellular internalization of CLDN4 protein in the presence of various endocytic inhibitors 45. The chlorpromazine and mβCD exerted an inhibitory effect on the decrease of CLDN4 protein for all particles (**Figure 5B and Figure S11**), indicating that the CLDN4 protein was endocytosed into endosomes via both the clathrin- and caveolae-mediated pathways. Additionally, PCB_23_/INS and PCB_36_/INS could induce macropinocytosis-dependent endocytosis as the wortmannin inhibited macropinocytosis by inhibiting phosphatidyl inositol-3-phosphate. Furthermore, CLSM was used to visualize the intracellular distribution of CLDN4. Cells were stained with LysoTracker deep red to label the endosomes/lysosomes. The CLDN4 on plasma membrane was destroyed by Triton-X-100. Compared with untreated control, the green fluorescence of CLDN4 in cells treated with particles was colocalized with red fluorescence of LysoTracker deep red as yellow dots (**Figure 5C and Figure S12**). These results confirmed that CLDN4 was decreased via the endocytosis-mediated lysosomal degradation pathway.

The transmembrane integral proteins were linked with F-actin cytoskeleton 40. To detect whether the PCB/INS particles stimulated the structural change of F-actin cytoskeleton, the F-actin of Caco-2 cell monolayers was stained with FITC-labeled phalloidin after particles treatment. The cells without particles treatment showed a regular fluorescence distribution of F-actin along the cell perimeter (**Figure 5D**). In comparison, depolymerization and actin stress fibers formed 2 h after PCB/INS particles treatment (**Figure 5D and Figure S13**). Therefore, we found that PCB/INS particles could induce the depolymerization of F-actin cytoskeleton, which was the important reason for the clathrin-, caveolae- and macropinocytosis-mediated lysosomal degradation pathway 46,47. Additionally, the structure of F-actin cytoskeleton gradually recovered after particles removal.

Furthermore, integrins have been recognized as the binding sites on cell membrane for anionic particles 20,23. To test this, Caco-2 cell monolayers were blocked with integrin αV antibody or integrin β1 antibody before and during particles incubation. The CLDN4 down-regulation effect was interfered except the integrin αV antibody for PCB_122_/INS particles (**Figure 5E and 5F**). Therefore, although the binding sites for particles with different morphologies varied, the integrins were critical for the particles binding process.

The PCB/INS particles were charge-switchable due to the protonation and deprotonation of carboxyl acid groups (**Figure 5G**). Under the pH 7.4 environment, the density of negatively charged carboxyl acid groups was increased and they were distributed on the surface of the particles due to their electrostatic repulsion with insulin. Therefore, the PCB/INS particles became anionic particles. On one hand, the anionic particles played an intestinal permeation enhancer role by binding with integrins to induce the depolymerization of F-actin cytoskeleton, which resulted in endocytosis-mediated lysosomal degradation pathway of the transmembrane integral proteins. On the other hand, insulin was gradually released from the particles due to the electrostatic repulsion. Free insulin was then absorbed into the bloodstream via the enhanced paracellular pathway. Importantly, this process was reversible without permanently destroying the cell monolayers.

### PCB_122_/INS particles enabled efficient treatment of type 1 diabetes mellitus

**Figure 6:**
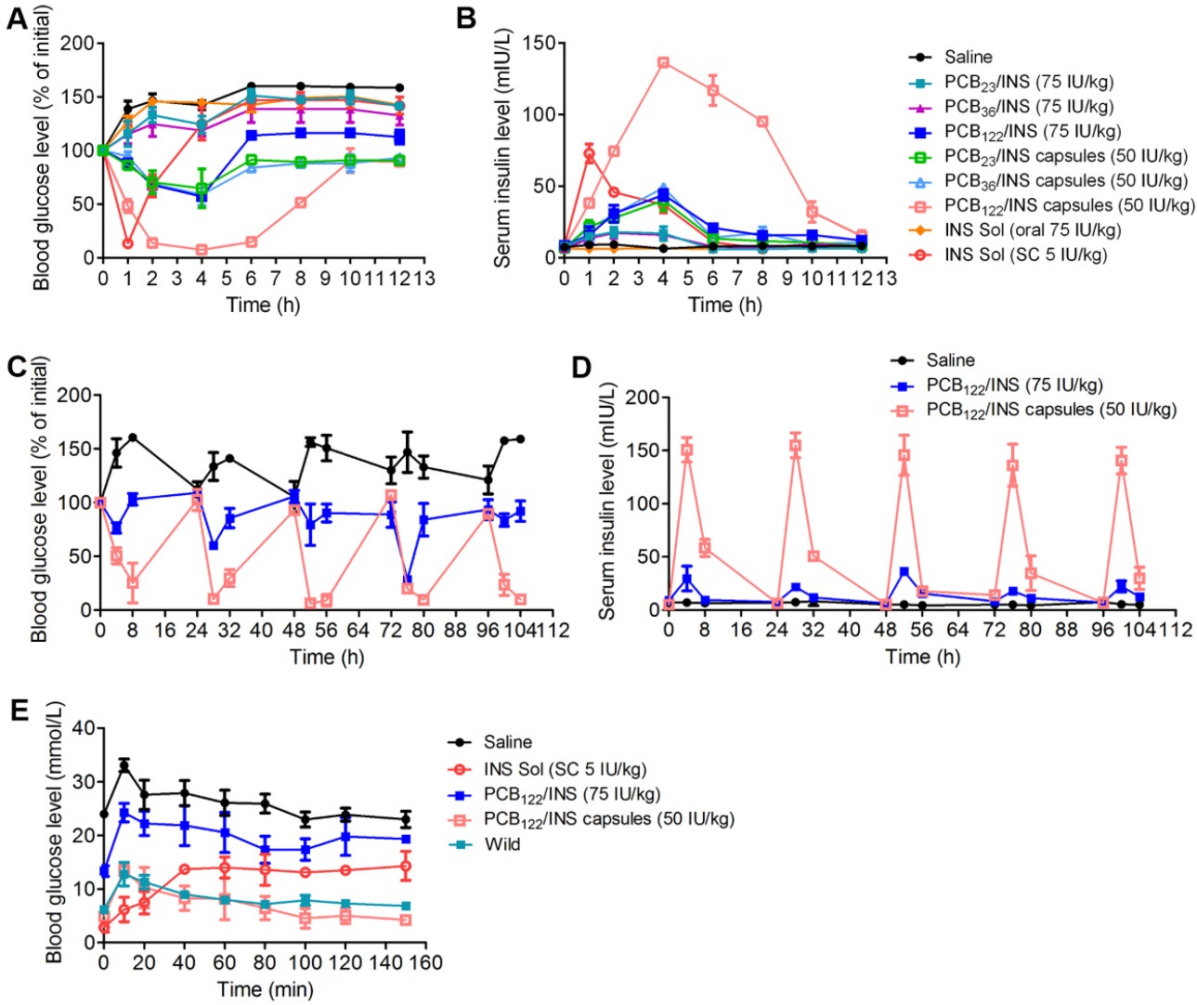
** PCB_122_/INS particles enabled oral insulin delivery in STZ-induced type 1 diabetic rats. (A)** Variation of blood glucose levels of diabetic rats after treatment for 12 h. **(B)** Variation of serum insulin level of diabetic rats after treatment for 12 h. Diabetic rats were orally administered PCB/INS particles, insulin solution at a dose of 75 IU/kg, PCB/INS particles in capsules at a dose of 50 IU/kg. Subcutaneous (SC) injection of insulin solution at a dose of 5 IU/kg, or saline via gavage. **(C)** Variation of blood glucose levels of diabetic rats after treatment. **(D)** Variation of serum insulin level of diabetic rats after treatment. Diabetic rats were orally administered PCB_122_/INS particles at a dose of 75 IU/kg and PCB_122_/INS particles in capsules at a dose of 50 IU/kg once a day for five consecutive days. **(E)** *In vivo* glucose tolerance test toward diabetic mice 4 h post-administration of PCB_122_/INS particles at a dose of 75 IU/kg or PCB_122_/INS particles in capsules at a dose of 50 IU/kg. The mean ± SD was shown (n = 5).

Finally, we evaluated the hypoglycemic effect and pharmacokinetics following oral administration of PCB/INS particles in streptozotocin (STZ)-induced type 1 diabetic rats. Similar to the saline, oral administration of free insulin solution failed to reduce the blood glucose level, while the oral administration of PCB_122_/INS particles generated about 42.7% of blood glucose level reduction at 4 h (**Figure 6A**). The blood glucose level of the PCB_122_/INS particles remained 114.0% and 112.6% of initial with free access to food and water at 6 and 12 h administration, respectively. In comparison, it was 146.9% and 141.8% for diabetic rats that were subcutaneous injected of insulin solution at 6 and 12 h, respectively. This might be attributed to the sustained release ability of PCB_122_/INS particles. Moreover, the hypoglycemic effect and pharmacokinetics of PCB_122_/INS particles were both better than the other two particles (**Figure 6A and 6B**). NPs in mucus were reported to transport primarily through low viscosity pores within the elastic, and the mess spacing ranges approximately from 10 to 200 nm 48. Therefore, the PCB_122_/INS particles with diameter less than 200 nm could better meet the sterical requirement for easy diffusion (**Figure 2F**).

However, the bioavailability of insulin in PCB_122_/INS particles was only 5.4%. The harsh stomach environment might seriously limited their therapeutic efficiency. Therefore, it was necessary to encapsulate the particles by entric coated capsules to overcome this barrier 8,18. We directly lyophilized the solution form of PCB/INS particles into powders without any additive cryoprotectant. This process had almost no influence on their size distribution and loading efficiency of insulin (**Figure S14**). We then prepared the entric coated capsules by directly packaging the PCB/INS particles powders into porcine gelatin capsule, followed by an Eudragit L100-55 enteric-coating procedure. The capsules significantly enhanced the pharmacological availability and bioavailability of insulin (**Figure S15**). PCB_122_/INS capsules exhibited the highest availability with approximately 27.0%. Therefore, the following studies were focused on the PCB_122_/INS particles and capsules.

Diabetes is a chronic disease and patients require insulin daily 49. To verify the therapeutic efficacy after multiple administration, diabetic rats were orally administered PCB_122_/INS particles at a dose of 75 IU/kg and PCB_122_/INS capsules at a dose of 50 IU/kg once a day for five consecutive days. As shown in **Figure 6C and 6D**, the blood glucose level of diabetic rats decreased after every oral administration, especially for the PCB_122_/INS capsules treatment group. The blood glucose level was gradually increased to the level before treatment at 24 h. Therefore, the capsules could be given once a day.

An intraperitoneal glucose tolerance test (IPGTT) was performed to evaluate the effectiveness of the particles associated with *in vivo* glucose response 4 h after treatment. As shown in **Figure 6E**, blood glucose peaks were observed for all groups with intraperitoneal glucose injection, while only the PCB_122_/INS capsules reestablished normoglycemia in a brief period as the healthy rats 50.

### The biocompatibility and safety of PCB_122_/INS particles

**Figure 7:**
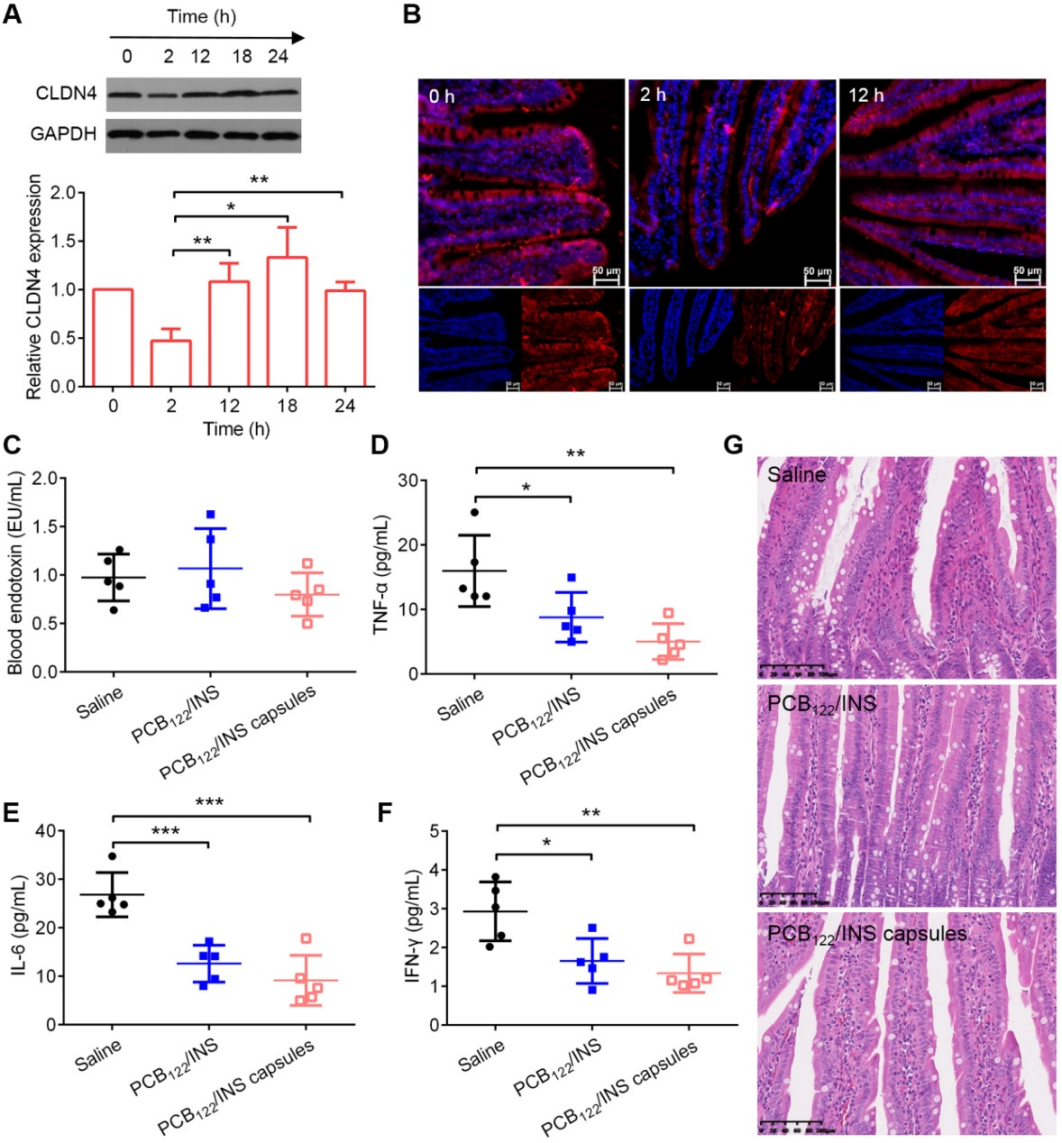
** The biocompatibility and safety of PCB_122_/INS particles. (A)** Western blot results of CLDN4 in small intestine of diabetic rats treated with PCB_122_/INS particles for 24 h. Western bands were scanned and normalized to the gray values of internal control GAPDH. The results were quantified using ImageJ. The data was relative to the untreated control in each Western band. The untreated control was settled to 1. The mean ± SD was shown (n = 3).** (B)** Fluorescence images of small intestines collected by CLSM. CLDN4 was shown in red, and cell nuclei stained with DAPI were in blue. The scale bar was 50 µm.** (C-F)** The endotoxin and pro-inflammatory cytokines in blood serum that was collected 24 h after the last administration.** (G)** Hematoxylin-eosin staining of small intestines. The scale bar was 100 µm. Diabetic rats were orally administered PCB_122_/INS particles at a dose of 75 IU/kg and PCB_122_/INS particles in capsules at a dose of 50 IU/kg once a day for five consecutive days. The mean ± SD was shown (n = 5). *P < 0.05, **P < 0.01, ***P < 0.001.

The changes of CLDN4 level after treatment were evaluated by Western blot. Western blot results showed that CLDN4 protein was reduced after 2 h of PCB_122_/INS particles treatment and recovered to the normal level after 12 h (**Figure 7A**). The results from immunofluorescence staining of small intestinal tissues were corresponding with this (**Figure 7B and Figure S16**). The endotoxin and pro-inflammatory cytokines were conducted to verify the long-term safety. PCB_122_/INS particles and capsules did not induce any observable increase in terms of endotoxin (**Figure 7C**). In addition, PCB_122_/INS particles and capsules could reduce the pro-inflammatory cytokines including IL-6, IFN-γ and TNF-α that contributed to the pathogenesis of STZ-induced diabetes (**Figure 7D-7F**) 51. Furthermore, to assess the integrity of intestinal tissues following treatment, hematoxylin-eosin staining was performed on the small intestines after five consecutive administration. As shown in **Figure 7G**, there was no inflammation and pathological changes during treatment, indicating that PCB_122_/INS particles were safe enough for *in vivo* application 52.

## Conclusion

Oral administration of protein drugs is considered to be the most convenient and safe choice for patients because of high patient compliance and repeatable administration. However, the bioavailability of oral protein drugs is very low due to the poor oral absorption into blood circulation. Although there have been many intestinal permeation enhancers existing, few of them have been approved for clinical application due to their poor dosage form assembling ability and high cytotoxicity. Charge-switchable zwitterionic PCB might offer a promising alternative choice. PCB loading with insulin via electrostatic interaction could induce the open of the tight junctions of intestinal epithelium in endocytosis-mediated lysosomal degradation pathway. Importantly, PCB/INS particles had almost no cytotoxicity. Their opening effect on the intestinal epithelium was reversible without causing lasting health problem, indicating that PCB/INS particles were safe enough for *in vivo* application. Furthermore, PCB/INS particles with diameter sub-200 nm, especially in capsules, enabled the oral delivery of insulin with bioavailability of 27.0% and achieved hypoglycemic effect for a few hours longer than subcutaneously injected insulin. Therefore, PCB/INS particles with diameter sub-200 nm could be used as an intestinal permeation enhancer for efficient type 1 diabetes mellitus treatment. Most importantly, this system has the potential to open up a new avenue for oral protein drugs administration for diseases treatment.

## Methods

### Materials

2-(N,N'-dimethylamino) ethyl methacrylate (DMAEMA, 98%) was purchased from Alfa Aesar. β-Propiolactone (98%) and 2-cyanoprop-2-yl-dithiobenzoate were from J&K Scientific Ltd. Porcine insulin, FITC-labeled insulin and trypsin were purchased from Dalian Meilun Biotechnology Co., Ltd. 2,2'-Dicyano-2,2'-azopropane (AIBN) was from Aladdin Industrial Corporation. MTT, FITC-labeled phalloidin, porcine stomach mucin and STZ were obtained from Sigma Aldrich. LysoTracker deep and Pierce^TM^ Coomassie Plus (Bradford) Assay Kit were purchased from Thermo Fisher Scientific. Alamar blue assay, 4% paraformaldehyde, normal goat serum and bafilomycin A1 were from Beijing Solarbio Science & Technology Co., Ltd. The Eudragit L 100-55 was from Shanghai Chineway Pharmaceutical Technology Co., Ltd. Triton® X-100 was from MP Biomedicals, LLC. Anti-claudin-4 antibody and Alexa-Fluor-conjugated secondary antibody were obtained from Abcam. Anti-integrin αV and β1 antibodies were purchased from Affinity Biosciences LTD. All plates used in cellular experiments were Corning Incorporated.

### Synthesis and purification of PCB

Carboxybetaine (CB) monomer was synthesized according to the method reported in our researches before 26-28. PCB with the theoretical DP of 50 was synthesized by the reversible addition-fragmentation chain transfer polymerization. 2-Cyanoprop-2-yl-dithiobenzoate (6 mg), CB (311.1 mg) and AIBN (1.5 mg) were dissolved in methanol, and were added to a clean and dry schlenk flask. The system was degrassed by three freeze-pump-thaw cycles and recharged with nitrogen. The reaction mixture was stirred for 24 h at 60 °C. The impurities and unreacted monomers were removed by dialyzing in a Cellu SepH1-membrane (MWCO 3,500) against ethanol and deionized water for 48 h, respectively. The final product was obtained by lyophilization. PCB with other DP was synthesized by the same method. ^1^H NMR (Bruker 600 MHz, D_2_O, δ ppm) was carried out to characterize the obtained product. Gel permeation chromatography was used to measure the molecular weight.

### Preparation and characterization of PCB/INS particles and capsules

PCB was dissolved in 0.1 M citrate buffer with pH 5.0 at a concentration of 2.0 mg/mL. Insulin was dissolved in 0.01 M NaOH at a concentration of 1.0 mg/mL. The insulin solution was added dropwise to the PCB solution at different mass ratios under stirring. The mixture was stirred at 37 °C for 10 min to yield PCB/INS particles. Furthermore, PCB/FITC-INS particles were prepared with the same method using FITC-labeled insulin. The obtained particles were collected by centrifugation at 8,000 rpm for 15 min. The collected particles were redispersed in 0.1 M citrate buffer with pH 6.0.

To prepare the oral insulin capsules, dry powders of PCB_23_/INS, PCB_36_/INS and PCB_122_/INS particles were obtained by lyophilization for 12 h after freezing at -20 °C. The dry powders were placed into bovine gelatin capsules (size 4#, GS Capsule Co., Ltd), respectively. The capsules were then immersed in the Eudragit L 100-55 methanol solution (15% w/w) and dried at room temperature three times to obtain the capsules with enteric coating 13. The thickness of the coating layer was 0.2223±0.0095 mm detected by vernier caliper.

The morphological analysis was carried out by TEM (JEM-1200EX). The zeta potential of particles was characterized with a Malvern Zetasize Nano ZS instrument. The distribution of FITC-insulin in particles was observed with DeltaVision OMX V3 imaging system (GE Healthcare). To determine the loading efficiency and loading content of insulin in particles, FITC-insulin in PCB_23_/INS, PCB_36_/INS, PCB_122_/INS particles was released by adding 10% methanol. The amount of FITC-insulin was assayed by microplate reader. The loading efficiency and loading content were calculated using the equations listed below, respectively.

Loading efficiency (%) = W_insulin in particles_/W_total insulin_ × 100%

Loading content (%) = W_insulin in particles_/W_total particles_ × 100%

### *In vitro* release and stability study

PCB_23_/FITC-INS, PCB_36_/FITC-INS and PCB_122_/FITC-INS particles in dialysis bag (MWCO 10,000) were incubated in 50 mL of distinct pH dissolution media at 37 °C under agitation, respectively. At pre-determined time intervals, 1.0 mL solution was removed and the same volume of fresh solution was added. The samples were measured by microplate reader. The release profiles were calculated using the equation list below.

Release profile (%) = W_total insulin in solution_/W_total insulin in particles_ × 100%

The stability of insulin released from the PCB_23_/INS, PCB_36_/INS and PCB_122_/INS particles was evaluated using the Jasco J-810 conformation by the CD spectrum. The free insulin solution with a 0.1 mg/mL concentration was measured as the standard CD spectrum. To separate the insulin from particles, 10% methanol was added and the mixture was centrifugated at 8,000 rpm for 15 min. The supernatant was collected for CD measurement.

PCB/INS particles and free insulin were incubated with trypsin solution (50 IU/mL) at a concentration of 0.2 mg insulin/mL. At determined time points, 100 μL of above solution was removed and 10 μL of HCl (0.1 M) was added to terminate the enzymatic reaction. 100 μL of DMSO was added to destroy the particles and the amount of insulin was measured by high performance liquid chromatography assay (HPLC). The HPLC was equipped with a UV detector and a C18 column at room temperature. Mobile phase with acetonitrile and 0.1% trifluoroacetic acid was used at a ratio of 30: 70 (v/v, acetonitrile: 0.1% trifluoroacetic acid). The ratio was linearly changed to 40: 60 (v/v) over 5 min. The flow rate was 1.0 mL/min. The concentration of insulin was determined at 214 nm. The percentage of insulin remained (%) was calculated using the following equation.

Insulin remained (%) = W_solution_/W_total_ × 100%

### Motion behavior in mucus

Mucus was prepared by dissolving porcine stomach mucin in PBS at 20 mg/mL. PCB_23_/FITC-INS, PCB_36_/FITC-INS and PCB_122_/FITC-INS particles were added into 500 μL of mucus to reach a final concentration of 10% v/v (1.8 × 10^-4^ w/v) in Petri dishes. The mucus with particles was equilibrated for 30 min before observation. Videos were captured with a × 64 oil-immersion objective in a CLSM (Leica SP8 STED 3X) and analyzed by Volocity.

### Cytotoxicity measurement

To determine the cytotoxicity of particles, Caco-2 cells were seeded in 96-well plates at a concentration of 5 × 10^3^ cells per well in 100 μL of DMEM containing 20% fetal bovine serum (FBS), 1% penicillin (100 units/mL) and streptomycin (100 μg/mL) at 37 °C under a 5% CO_2_ atmosphere for 24 h. PCB_23_/INS, PCB_36_/INS, PCB_122_/INS particles with different concentrations of PCB were then added to each well with 100 μL of the culture medium, respectively. After 24 h incubation of particles with cells, 20 μL of MTT solution (5 mg/mL in PBS) was added to each well and incubated for additional 2 h. The medium was then replaced with 100 μL of DMSO. The absorbance was measured at 562 nm using a Tecan microplate reader (Tecan, Switzerland). Additionally, Alarma blue assay was utilized to evaluate the cell proliferation. Untreated Caco-2 cells were used as control and their cell viability was defined as 100%.

### Transport of PCB/INS particles across Caco-2 cell monolayer

Caco-2 cells were cultured in 24-well transwell^®^ plates and used when resistance across the insert membrane ranged from 500 to 700 Ω cm^2^. The apical and basolateral sides of transwell^®^ were changed to HBSS buffer at pH 7.4 with 10% FBS. PCB_23_/FITC-INS, PCB_36_/FITC-INS and PCB_122_/FITC-INS particles at a concentration of 5 μg PCB/mL were added to the apical buffer, respectively. After that, the basolateral buffer was collected at 24 h, and the fluorescence intensity of FITC-insulin was measured using a Tecan microplate reader to calculate the FITC-insulin concentration according to the standard curve. *P*_app_ was calculated using the following equation. The dQ/dt was the amount of insulin transport to basolateral side per second. A was the diffusion area of the monolayer (0.33 cm^2^), and C_0_ was the initial insulin concentration.

*P*_app_ = (dQ/dt) × 1/(AC_0_)

With the same condition, the TEER values were measured by the resistance test instrument (Millicell ERS-2, Millipore) at designed time point after PCB_23_/INS, PCB_36_/INS and PCB_122_/INS particles addition or removing in 24-well transwell^®^ plates. The changes in the TEER against time were plotted.

### Distribution of FITC-insulin

Caco-2 cells were grown in Petri dishes and then treated with PCB_23_/FITC-INS, PCB_36_/FITC-INS and PCB_122_/FITC-INS particles at a concentration of 5 μg PCB/mL, respectively. Following incubation for 5 h, particles were aspirated and cells were then gently washed three times with PBS. After fixation with 4% paraformaldehyde for 10 min, the nuclei were stained with DAPI for 10 min. After three times washing with PBS, the images of the cell monolayers were obtained using an inverted CLSM.

At the same time, cells were grown in 12-well plates and treated with PCB_23_/FITC-INS, PCB_36_/FITC-INS and PCB_122_/FITC-INS particles of 5 μg PCB/mL for 5 h, respectively. Cells were washed three times with PBS, digested with trypsin and harvested in PBS. The samples were assessed with BD Caliburflow cytometry (BD CO., USA) to determine the mean fluorescence intensity of FITC.

### Western blotting and real-time PCR analysis

Caco-2 cells were seeded in 6-well plates and treated with PCB_23_/INS, PCB_36_/INS, PCB_122_/INS particles at a concentration of 5 μg PCB/mL for 2 h, respectively. Cells were then washed with PBS three times and the level of CLDN4 mRNA was assayed using real-time PCR analysis.

Western blot was used to determine the level of CLDN4 protein. Caco-2 cells were seeded in 6-well plates at a concentration of 5 × 10^5^ cells per well for 3 days. Cells were treated with PCB_23_/INS, PCB_36_/INS, PCB_122_/INS particles at a concentration of 1 μg PCB/mL or 5 μg PCB/mL for 2 h, respectively. Cells were then gently washed three times with PBS and lysated with a lysis buffer.

To determine the recovery of the CLDN4 protein, Caco-2 cells were seeded in 6-well plates and treated with PCB_23_/INS, PCB_36_/INS, PCB_122_/INS particles at a concentration of 1 μg PCB/mL or 5 μg PCB/mL for 2 h, respectively. Cells were washed with PBS three times and cultured for another 2 h with fresh culture medium. Cells were then gently washed three times with PBS and lysated with a lysis buffer.

To investigate the autophagy influence, Caco-2 cells were incubated with 200 nM bafilomycin A1 for 30 min prior to the transfection, and the same concentration of bafilomycin A1 was added to the medium during culturing with PCB_23_/INS, PCB_36_/INS, PCB_122_/INS particles at a concentration of 5 μg PCB/mL for 2 h, respectively. Cells were gently washed three times with PBS and lysated with a lysis buffer.

For the integrin blockade, Caco-2 monolayers in 6-well plates were incubated for 1 h with 25 μg/mL of the anti-integrin αV and β1 antibodies, respectively 23. Cells were cultured with PCB_23_/INS, PCB_36_/INS and PCB_122_/INS particles at a concentration of 5 μg PCB/mL for 2 h with the same concentration of anti-integrin αV and β1 antibodies. Cells were gently washed three times with PBS and lysated with a lysis buffer. Subsequently, the lysis buffers were centrifuged at 10,000 rpm for 5 min, and the supernatant was collected for the subsequent Western blot analysis. The protein concentration was calculated using the Bradford protein assay kit.

### Immunofluorescence staining

For immunofluorescence staining, Caco-2 cells were grown in Petri dishes and treated with PCB_23_/INS, PCB_36_/INS, PCB_122_/INS particles at a concentration of 5 μg PCB/mL for 2 h, respectively. Cells were washed with PBS three times and cultured for another 2 h with fresh culture medium. The treated cells were washed three times with PBS and fixed with 4% paraformaldehyde for 10 min. Non-specific binding was blocked in 10% normal goat serum in PBS for 1 h. Cells were then incubated with the primary anti-claudin-4 antibody (5 μg/mL) overnight at 4 °C. After washing, the Alexa-Fluor-conjugated secondary antibody was incubated for 1 h. The cell nuclei were stained with DAPI for 10 min. After washing, cells were observed using CLSM.

To observe the intracellular accumulation of CLDN4, Caco-2 cells were grown in Petri dishes and then treated with PCB_23_/INS, PCB_36_/INS, PCB_122_/INS particles at a concentration of 5 μg PCB/mL for 2 h, respectively. Cells were washed three times with PBS and followed by staining with LysoTracker deep red for 30 min at 37 °C. Cells were washed three times and fixed with 4% paraformaldehyde for 10 min. Cells were then treated with 0.3% Triton-X-100 for 30 min at room temperature. After that, CLDN4 and cell nuclei were stained as the procedures above. Cells were observed using CLSM.

To observe the F-actin cytoskeleton, Caco-2 cells were grown in Petri dishes and then treated with PCB_23_/INS, PCB_36_/INS, PCB_122_/INS particles at a concentration of 5 μg PCB/mL for 2 h, respectively. Cells were washed with PBS three times and cultured for another 2 h with fresh culture medium. Cells were washed three times with PBS and fixed with 4% paraformaldehyde for 10 min. The cells were treated with 0.1% Triton X-100 for 10 min at room temperature. FITC-labeled phalloidin (50 μg/mL) was then added for 1 h at room temperature, and the cell nuclei were stained with DAPI for 10 min. Cells were then washed three times with PBS, and visualized on a CLSM.

### Hypoglycemic effect and pharmacokinetic

The hypoglycemic effect and pharmacokinetic of the particles following oral administration were evaluated on diabetic rats. All procedures were performed in accordance with guidelines approved by the ethics committee of Tsing Hua University. For the disease induction, male Sprague-Dawley rats weighting 180-220 g were injected with STZ (65 mg/kg) once via the intraperitoneal injection. Blood glucose level was determined using a glucose meter. The rats that exhibited fasting blood glucose level over 16.0 mM three days after injection were considered to be diabetic. Saline, free insulin solution at a dose of 75 IU/kg, PCB_23_/INS, PCB_36_/INS and PCB_122_/INS particles at a dose of 75 IU/kg were administered via gavage, respectively. One group of diabetic rats were subcutaneous injection with insulin solution at a dose of 5 IU/kg. PCB_23_/INS, PCB_36_/INS and PCB_122_/INS capsules at a dose of 50 IU/kg were oral administered using rat capsules dosing device. The rats were allowed free access to food and water 30 min after administration, and the experiments were started in the morning 53. Blood samples were collected and blood glucose level was determined using a glucose meter. Plasma insulin levels were quantified using the insulin ELISA kit (abcam). The pharmacological availability (PA%) and bioavailability (*F*%) of insulin relative to subcutaneous injection were calculated using the following equations. The area above the curve (AAC) was calculated from the blood glucose level curve. The area under the curve (AUC) was calculated from the plasma insulin concentration curve. At the same condition of oral administration of PCB_122_/INS particles, rats were euthanized and small intestines were harvested at designed time point. Western blot was performed to detect the level of CLDN4. Immunostaining of CLDN4 was performed and observed using CLSM.

PA (%) = (AAC_oral_ × Dose_SC_)/(AAC_SC_ × Dose_oral_) × 100%

*F* (%) = (AUC_oral_ × Dose_SC_)/(AUC_SC_ × Dose_oral_) × 100%

To verify the long-term therapeutic efficacy and safety, STZ-induced diabetic rats were orally administered of PCB_122_/INS particles at a dose of 75 IU/kg and PCB_122_/INS particles in capsules at a dose of 50 IU/kg once a day for five consecutive days. Blood samples were collected and blood glucose level was determined using a glucose meter. Plasma insulin levels were quantified using the insulin ELISA kit (abcam). Blood samples were collected to detect the endotoxin and pro-inflammatory cytokines 24 h after the last administration. The small intestines were also collected for hematoxylin-eosin staining 24 h after the last administration.

After 14 h fasting, STZ-induced diabetic rats were treated with orally administered saline, PCB_122_/INS particles at a dose of 75 IU/kg and capsules at a dose of 50 IU/kg, respectively. One group of diabetic rats was subcutaneous injection with insulin solution at a dose of 5 IU/kg. Glucose was intraperitoneal injected 4 h after treatment at a dose of 1.5 g/kg. Normal rats were used as control. Blood samples were collected at predetermined time points and blood glucose level was determined using a glucose meter.

### Statistical analysis

All data are expressed as mean ± SD unless otherwise indicated. Statistical significance was analyzed using one-way ANOVA. *p* values < 0.05 were considered statistically significant.

## Supplementary Material

Supplementary figures.

## Abbreviations

PCB: Polycarboxybetaine
PCB/INS: PCB/insulin
DP: degree of polymerization
TEM: transmission electron microscopy
CD: circular dichroism
MTT: 3-(4,5-dimethylthiazol-2-yl)-2,5-diphenyltetrazolium bromide
*P*_app_: apparent permeability coefficient
TEER: transepithelial electrical resistance
CLDN4: claudin-4
SC: subcutaneous
CB: carboxybetaine
IPGTT: intraperitoneal glucose tolerance test
STZ: streptozotocin
PA: pharmacological availability
*F*: bioavailability
AAC: area above the curve
AUC: area under the curve
HPLC: high performance liquid chromatography assay

## References

[1] Avram R, Olgin JE, Kuhar P, Weston Hughes J, Marcus GM, Pletcher MJ (2020). A digital biomarker of diabetes from smartphone-based vascular signals. Nat Med.

[2] Shan W, Zhu X, Liu M, Li L, Zhong J, Sun W (2015). Overcoming the diffusion barrier of mucus and absorption barrier of epithelium by self-assembled nanoparticles for oral delivery of insulin. ACS Nano.

[3] Zhu X, Wu J, Shan W, Zhou Z, Liu M, Huang Y (2016). Sub-50 nm nanoparticles with biomimetic surfaces to sequentially overcome the mucosal diffusion barrier and the epithelial absorption barrier. Adv Funct Mater.

[4] Brown TD, Whitehead KA, Mitragotri S (2019). Materials for oral delivery of proteins and peptides. Nat Rev Mater.

[5] Yu J, Qian C, Zhang Y, Cui Z, Zhu Y, Shen Q (2017). Hypoxia and H_2_O_2_ dual-sensitive vesicles for enhanced glucose-responsive insulin delivery. Nano Lett.

[6] Tauschmann M, Hovorka R (2018). Technology in the management of type 1 diabetes mellitus-current status and future prospects. Nat Rev Endocrinol.

[7] Chen Z, Wang J, Sun W, Archibong E, Kahkoska AR, Zhang X (2018). Synthetic beta cells for fusion-mediated dynamic insulin secretion. Nat Chem Biol.

[8] Sonaje K, Chen YJ, Chen HL, Wey SP, Juang JH, Nguyen HN (2010). Enteric-coated capsules filled with freeze-dried chitosan/poly(gamma-glutamic acid) nanoparticles for oral insulin delivery. Biomaterials.

[9] Sharma G, Sharma AR, Nam JS, Doss GP, Lee SS, Chakraborty C (2015). Nanoparticle based insulin delivery system: the next generation efficient therapy for type 1 diabetes. J Nanobiotechnology.

[10] Lin PY, Chiu YL, Huang JH, Chuang EY, Mi FL, Lin KJ (2018). Oral nonviral gene delivery for chronic protein replacement therapy. Adv Sci.

[11] Griffin BT, Guo J, Presas E, Donovan MD, Alonso MJ, O'Driscoll CM (2016). Pharmacokinetic, pharmacodynamic and biodistribution following oral administration of nanocarriers containing peptide and protein drugs. Adv Drug Deliv Rev.

[12] Shrestha N, Shahbazi MA, Araujo F, Zhang H, Makila EM, Kauppila J (2014). Chitosan-modified porous silicon microparticles for enhanced permeability of insulin across intestinal cell monolayers. Biomaterials.

[13] Han X, Lu Y, Xie J, Zhang E, Zhu H, Du H (2020). Zwitterionic micelles efficiently deliver oral insulin without opening tight junctions. Nat Nanotechnol.

[14] Sonaje K, Lin KJ, Wang JJ, Mi FL, Chen CT, Juang JH (2010). Self-assembled pH-sensitive nanoparticles: a platform for oral delivery of protein drugs. Adv Funct Mater.

[15] Hsu LW, Lee PL, Chen CT, Mi FL, Juang JH, Hwang SM (2012). Elucidating the signaling mechanism of an epithelial tight-junction opening induced by chitosan. Biomaterials.

[16] Zhang J, Zhu X, Jin Y, Shan W, Huang Y (2014). Mechanism study of cellular uptake and tight junction opening mediated by goblet cell-specific trimethyl chitosan nanoparticles. Mol Pharm.

[17] Yeh TH, Hsu LW, Tseng MT, Lee PL, Sonjae K, Ho YC (2011). Mechanism and consequence of chitosan-mediated reversible epithelial tight junction opening. Biomaterials.

[18] Sung HW, Sonaje K, Liao ZX, Hsu LW, Chuang EY (2012). pH-Responsive nanoparticles shelled with chitosan for oral delivery of insulin: from mechanism to therapeutic applications. Acc Chem Res.

[19] Kong XD, Moriya J, Carle V, Pojer F, Abriata LA, Deyle K (2020). De novo development of proteolytically resistant therapeutic peptides for oral administration. Nat Biomed Eng.

[20] Hsu LW, Ho YC, Chuang EY, Chen CT, Juang JH, Su FY (2013). Effects of pH on molecular mechanisms of chitosan-integrin interactions and resulting tight-junction disruptions. Biomaterials.

[21] Maher S, Mrsny RJ, Brayden DJ (2016). Intestinal permeation enhancers for oral peptide delivery. Adv Drug Deliv Rev.

[22] McCartney F, Jannin V, Chevrier S, Boulghobra H, Hristov DR, Ritter N (2019). Labrasol(R) is an efficacious intestinal permeation enhancer across rat intestine: *ex vivo* and *in vivo* rat studies. J Control Release.

[23] Lamson NG, Berger A, Fein KC, Whitehead KA (2020). Anionic nanoparticles enable the oral delivery of proteins by enhancing intestinal permeability. Nat Biomed Eng.

[24] McCartney F, Rosa M, Brayden DJ (2019). Evaluation of sucrose laurate as an intestinal permeation enhancer for macromolecules: *ex vivo* and *in vivo* studies. Pharmaceutics.

[25] Twarog C, Fattah S, Heade J, Maher S, Fattal E, Brayden DJ (2019). Intestinal permeation enhancers for oral delivery of macromolecules: a comparison between salcaprozate sodium (SNAC) and sodium caprate (C_10_). Pharmaceutics.

[26] Li Y, Cheng Q, Jiang Q, Huang Y, Liu H, Zhao Y (2014). Enhanced endosomal/lysosomal escape by distearoyl phosphoethanolamine-polycarboxybetaine lipid for systemic delivery of siRNA. J Control Release.

[27] Li Y, Li Y, Ji W, Lu Z, Liu L, Shi Y (2018). Positively charged polyprodrug amphiphiles with enhanced drug loading and reactive oxygen species-responsive release ability for traceable synergistic therapy. J Am Chem Soc.

[28] Zhang R, Li Y, Hu B, Lu Z, Zhang J, Zhang X (2016). Traceable nanoparticle delivery of small interfering RNA and retinoic acid with temporally release ability to control neural stem cell differentiation for Alzheimer's disease therapy. Adv Mater.

[29] Jeworrek C, Hollmann O, Steitz R, Winter R, Czeslik C (2009). Interaction of IAPP and insulin with model interfaces studied using neutron reflectometry. Biophys J.

[30] Tokumoto S, Higo N, Sugibayashi K (2006). Effect of electroporation and pH on the iontophoretic transdermal delivery of human insulin. Int J Pharm.

[31] Lin YH, Mi FL, Chen CT, Chang WC, Peng SF, Liang HF (2007). Preparation and characterization of nanoparticles shelled with chitosan for oral insulin delivery. Biomacromolecules.

[32] Wang A, Yang T, Fan W, Yang Y, Zhu Q, Guo S (2019). Protein corona liposomes achieve efficient oral insulin delivery by overcoming mucus and epithelial barriers. Adv Healthc Mater.

[33] Hu X, Yu J, Qian C, Lu Y, Kahkoska AR, Xie Z (2017). H_2_O_2_-responsive vesicles integrated with transcutaneous patches for glucose-mediated insulin delivery. ACS Nano.

[34] Johansson M, Sjövall H, Hansson G (2013). The gastrointestinal mucus system in health and disease. Nat Rev Gastroenterol Hepatol.

[35] Pereira de Sousa I, Steiner C, Schmutzler M, Wilcox MD, Veldhuis GJ, Pearson JP (2015). Mucus permeating carriers: formulation and characterization of highly densely charged nanoparticles. Eur J Pharm Biopharm.

[36] Ensign LM, Cone R, Hanes J (2012). Oral drug delivery with polymeric nanoparticles: the gastrointestinal mucus barriers. Adv Drug Deliv Rev.

[37] Qi J, Zhuang J, Lv Y, Lu Y, Wu W (2018). Exploiting or overcoming the dome trap for enhanced oral administration and drug delivery. J Control Release.

[38] Wu L, Liu M, Shan W, Zhu X, Li L, Zhang Z (2017). Bioinspired butyrate-functionalized nanovehicles for targeted oral delivery of biomacromolecular drugs. J Control Release.

[39] Drucker DJ (2020). Advances in oral peptide therapeutics. Nat Rev Drug Discov.

[40] Cong X, Zhang Y, Li J, Mei M, Ding C, Xiang RL (2015). Claudin-4 is required for modulation of paracellular permeability by muscarinic acetylcholine receptor in epithelial cells. J Cell Sci.

[41] Li X, Uehara S, Sawangrat K, Morishita M, Kusamori K, Katsumi H (2018). Improvement of intestinal absorption of curcumin by cyclodextrins and the mechanisms underlying absorption enhancement. Int J Pharm.

[42] Lee B, Moon KM, Kim CY (2018). Tight junction in the intestinal epithelium: its association with diseases and regulation by phytochemicals. J Immunol Res.

[43] Garcia-Hernandez V, Quiros M, Nusrat A (2017). Intestinal epithelial claudins: expression and regulation in homeostasis and inflammation. Ann N Y Acad Sci.

[44] Yu T, Liu X, Bolcato-Bellemin AL, Wang Y, Liu C, Erbacher P (2012). An amphiphilic dendrimer for effective delivery of small interfering RNA and gene silencing *in vitro* and *in vivo*. Angew Chem Int Ed Engl.

[45] Yin L, Song Z, Kim KH, Zheng N, Tang H, Lu H (2013). Reconfiguring the architectures of cationic helical polypeptides to control non-viral gene delivery. Biomaterials.

[46] Zhang Y, Zhang L, Li Y, Sun S, Tan H (2014). Different contributions of clathrin- and caveolae-mediated endocytosis of vascular endothelial cadherin to lipopolysaccharide-induced vascular hyperpermeability. PLoS One.

[47] Kabayama H, Nakamura T, Takeuchi M, Iwasaki H, Taniguchi M, Tokushige N (2009). Ca^2+^ induces micropinocytosis via F-actin depolymerization during growth cone collapse. Mol Cell Neurosci.

[48] Lai SK, Wang YY, Hanes J (2009). Mucus-penetrating nanoparticles for drug and gene delivery to mucosal tissues. Adv Drug Deliv Rev.

[49] Mansoor S, Kondiah PPD, Choonara YE, Pillay V (2019). Polymer-based nanoparticle strategies for insulin delivery. Polymers.

[50] Wang J, Ye Y, Yu J, Kahkoska AR, Zhang X, Wang C (2018). Core-shell microneedle gel for self-regulated insulin delivery. ACS Nano.

[51] Soetikno V, Sari FR, Veeraveedu PT, Thandavarayan RA, Harima M, Sukumaran V (2011). Curcumin ameliorates macrophage infiltration by inhibiting NF-κB activation and proinflammatory cytokines in streptozotocin induced-diabetic nephropathy. Nutr Metab.

[52] Volpatti LR, Matranga MA, Cortinas AB, Delcassian D, Daniel KB, Langer R (2020). Glucose-responsive nanoparticles for rapid and extended self-regulated insulin delivery. ACS Nano.

[53] Wang J, Yu J, Zhang Y, Zhang X, Kahkoska AR, Chen G (2019). Charge-switchable polymeric complex for glucose-responsive insulin delivery in mice and pigs. Sci Adv.

