# Supplementary material for: Charge-switchable zwitterionic polycarboxybetaine particle as an intestinal permeation enhancer for efficient oral insulin delivery
Source: Theranostics. 2021 Mar 4;11(9):4452–66. doi: 10.7150/thno.54176 (PMC7977443; doi:10.7150/thno.54176)
Supplement: Supplementary file 1 — Supplementary figures. [file thnov11p4452s1.pdf]

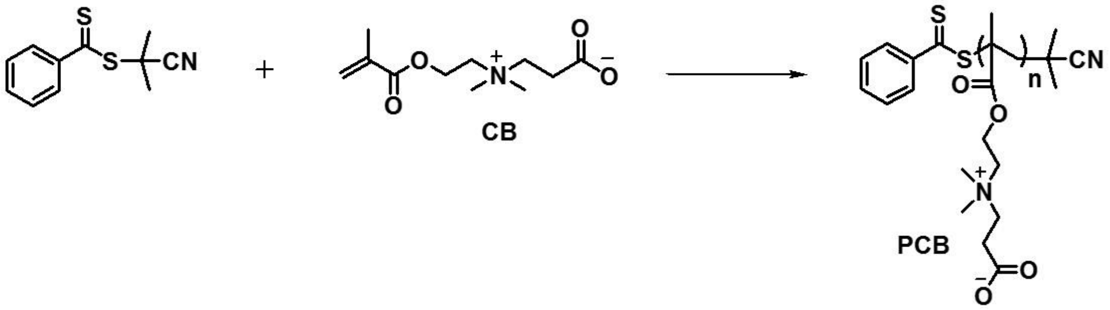

Figure S1. The synthetic route of PCB. PCB was synthesized via the reversible addition-fragmentation chain transfer polymerization.

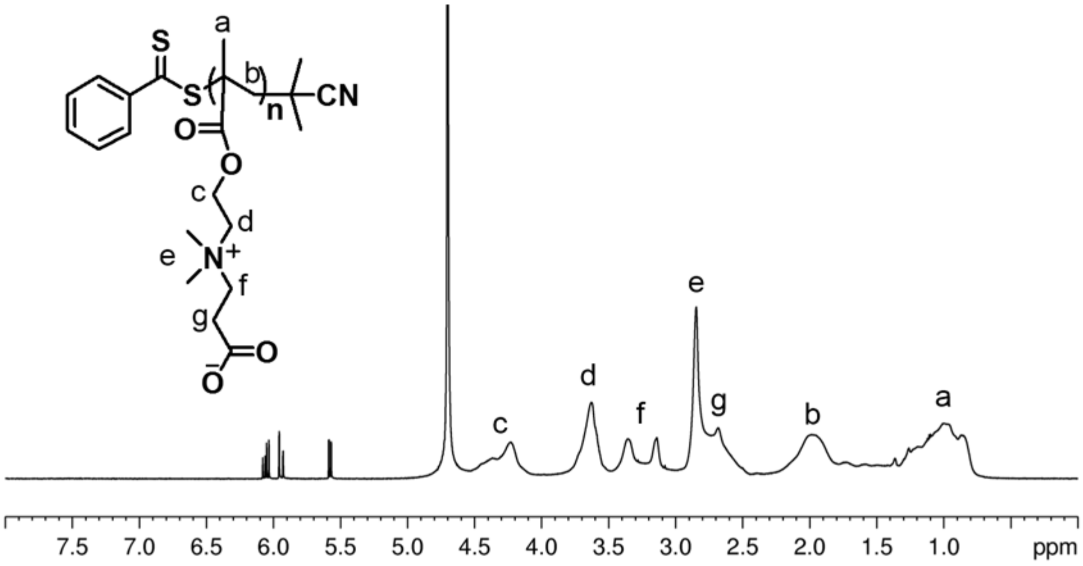

Figure S2. ^{1}H NMR characterization of PCB. ^{1}H NMR spectrum of PCB confirmed its successful synthesis.

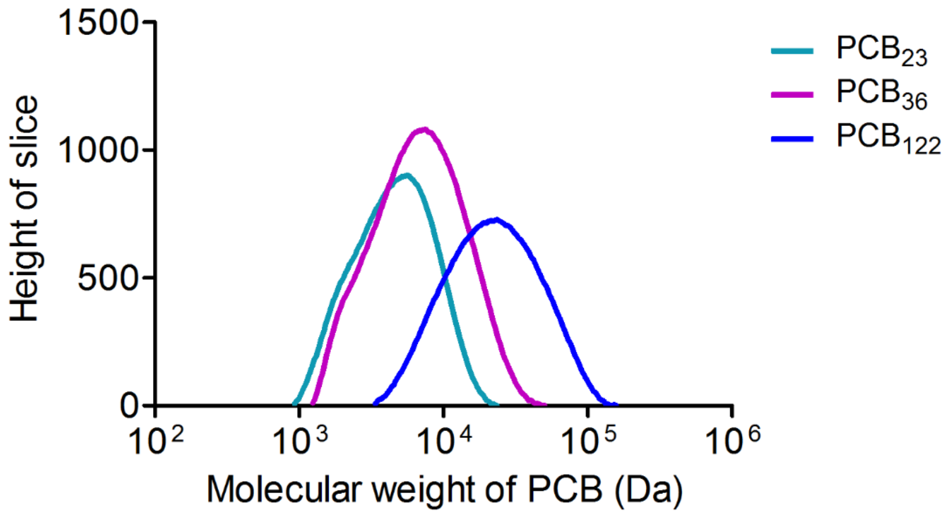

Figure S3. The molecular weight of PCB. The molecular weight slices of PCB were measured via gel permeation chromatography.

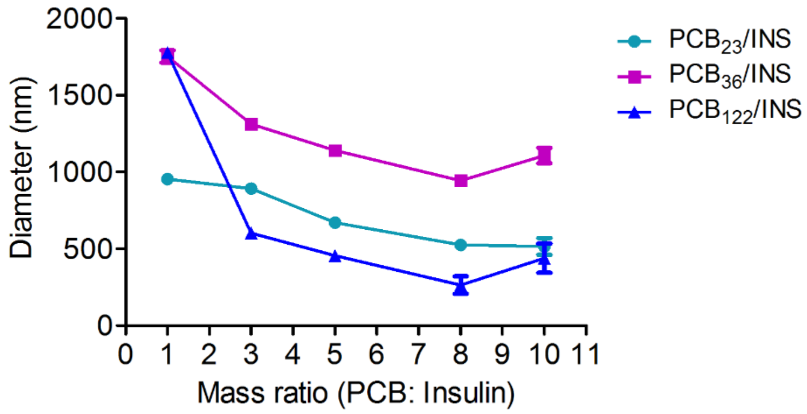

Figure S4. The diameter of particles at different mass ratios between PCB and insulin detected by dynamic light scattering. The mean ± SD was shown (n = 3).

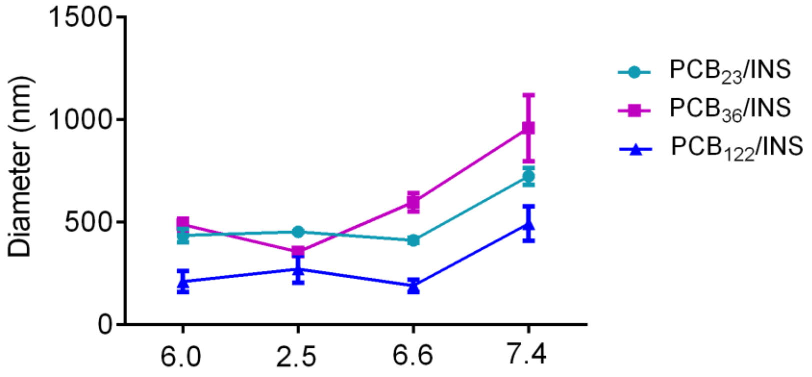

Figure S5. The diameter of particles at different pH microenvironments. The mean ± SD was shown (n = 3).

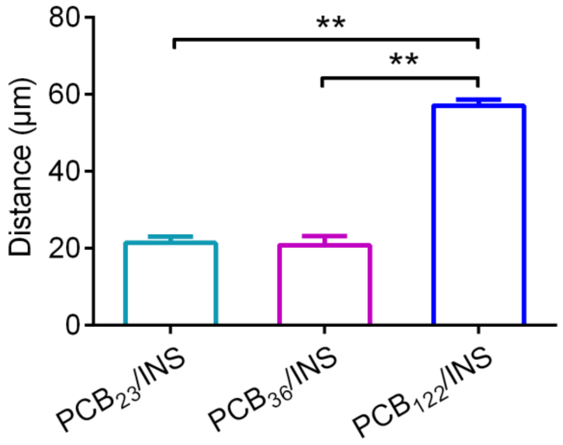

Figure S6. The mean distance of the PCB_{23}/INS, PCB_{36}/INS and PCB_{122}/INS particles in mucus at a time lapse of 7 s. The mean ± SD was shown (n = 3). $^{**}P < 0.01$.

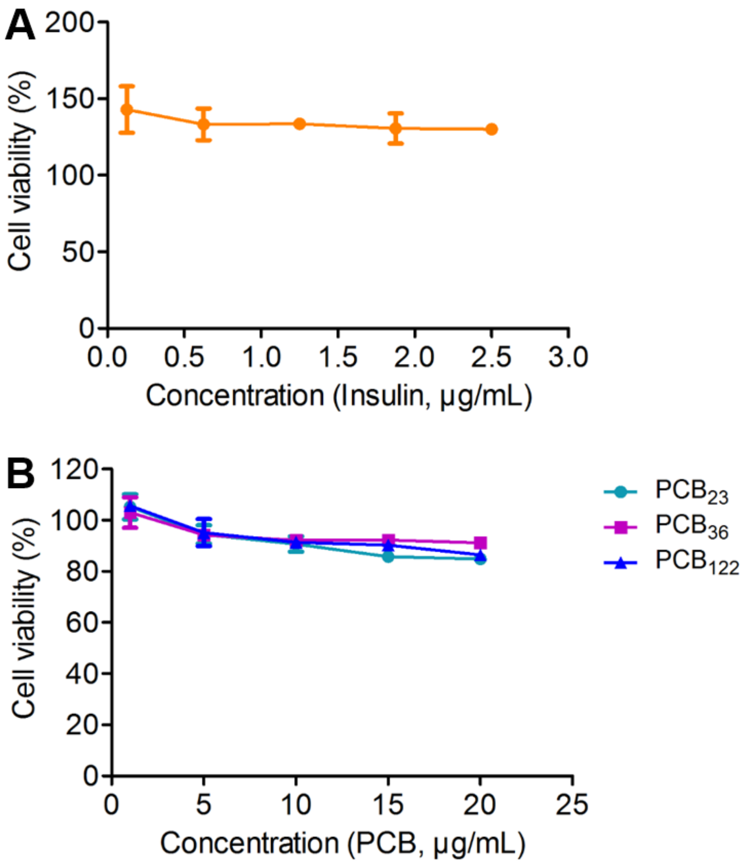

Figure S7. The cell viability of Caco-2 cells. **(A)** Cells were treated with different concentration of free insulin for 24 h. **(B)** Cells were treated with different concentration of PCB for 24 h. The cell viability was assessed using MTT assay. The mean ± SD was shown (n = 3).

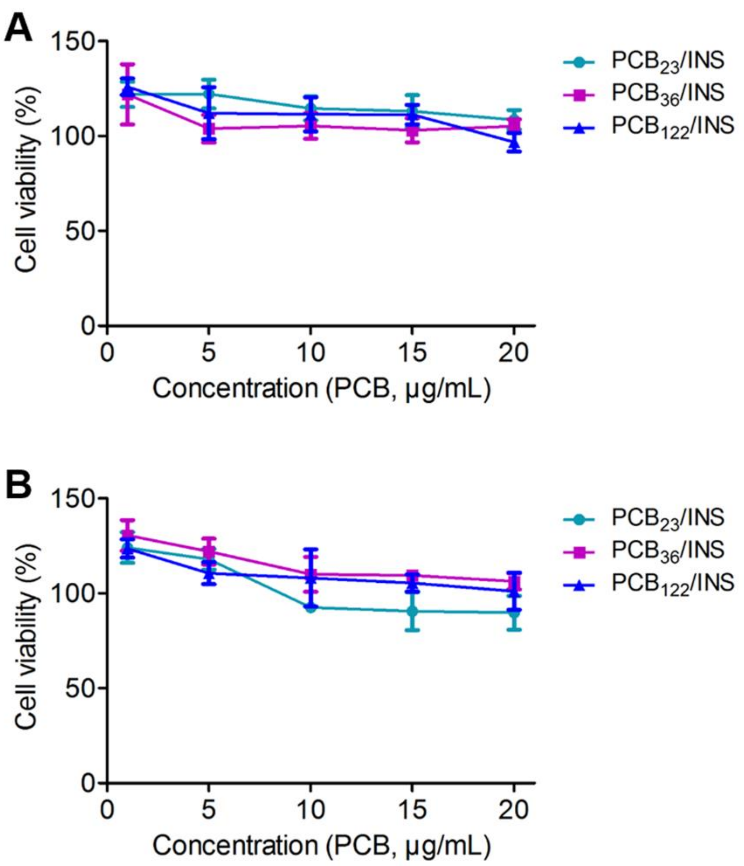

Figure S8. The cell viability of Caco-2 cells. Cells were treated with PCB/INS particles with different concentration of PCB for 24 h. The cell viability was assessed using **(A)** Alamar blue assay and **(B)** MTT assay. The mean ± SD was shown (n = 3).

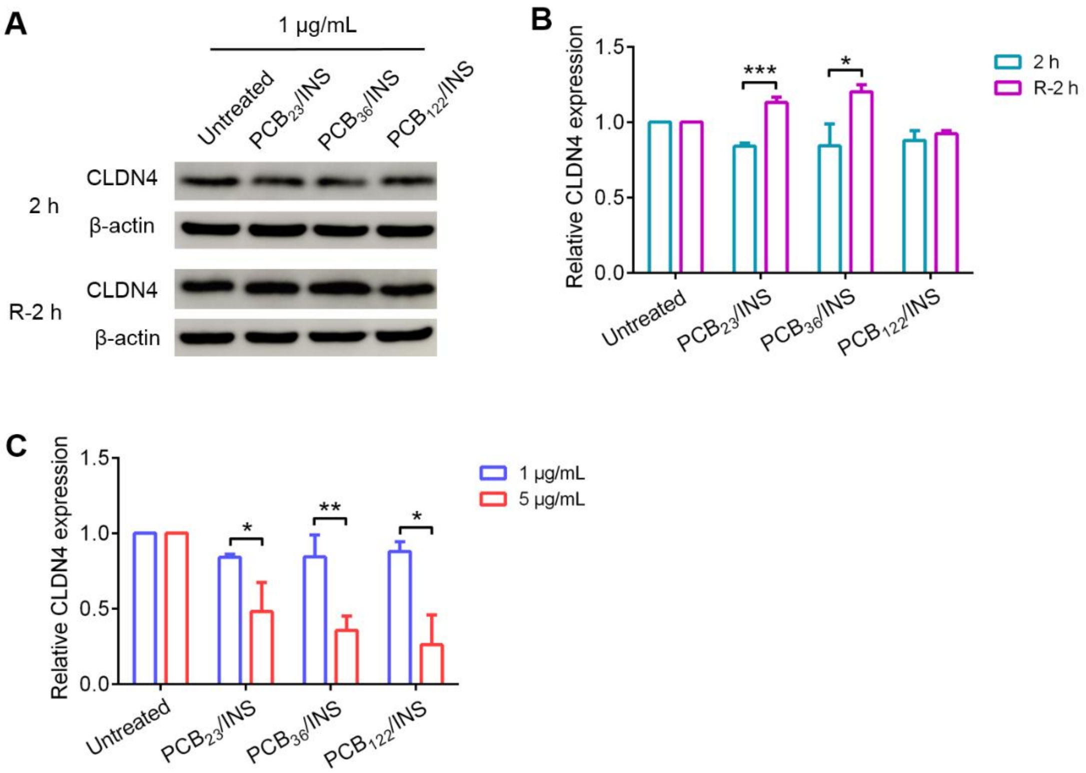

Figure S9. Western blot images and quantified results of CLDN4 protein after 2 h of PCB_{23}/INS, PCB_{36}/INS and PCB_{122}/INS particles incubation or removal. **(A,B)** Particles were at concentration of 1 µg PCB/mL. **(C)** Western blot quantified results of CLDN4 protein after 2 h of PCB_{23}/INS, PCB_{36}/INS and PCB_{122}/INS particles incubation at concentration of 1 µg PCB/mL and 5 µg PCB/mL, respectively. Western bands were scanned and normalized to the gray values of internal control β-actin. The results were quantified using ImageJ. The data was relative to the untreated control in each Western band. The untreated control was settled to 1. The mean ± SD was shown (n = 3). *$P < 0.05$, **$P < 0.01$, ***$P < 0.001$.

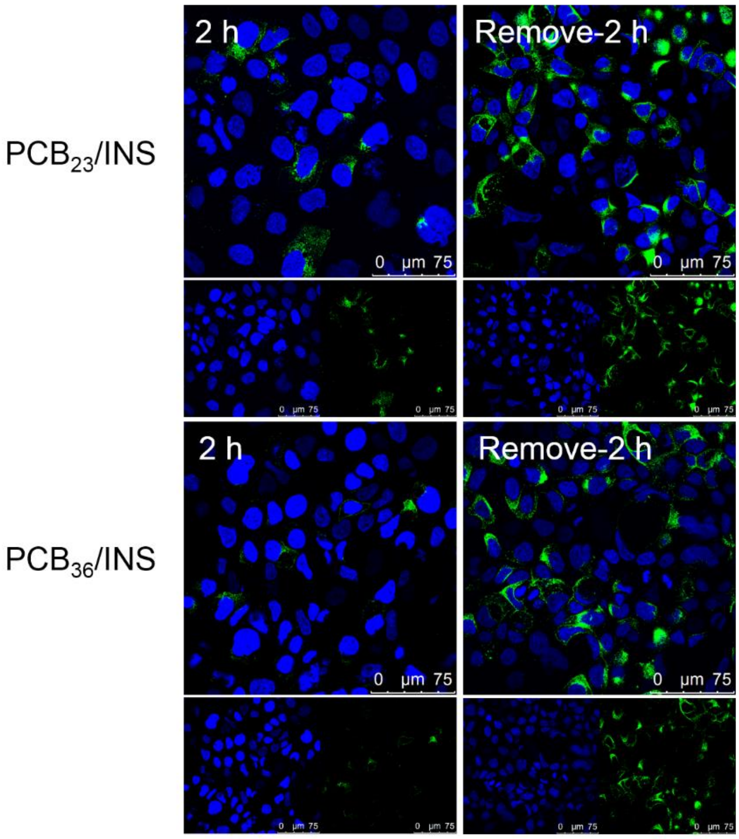

Figure S10. Fluorescence images collected by confocal laser scanning microscope (CLSM) after 2 h of PCB_{23}/INS or PCB_{36}/INS particles treatment or removal. CLDN4 was shown in green, and cell nuclei stained with DAPI were in blue.

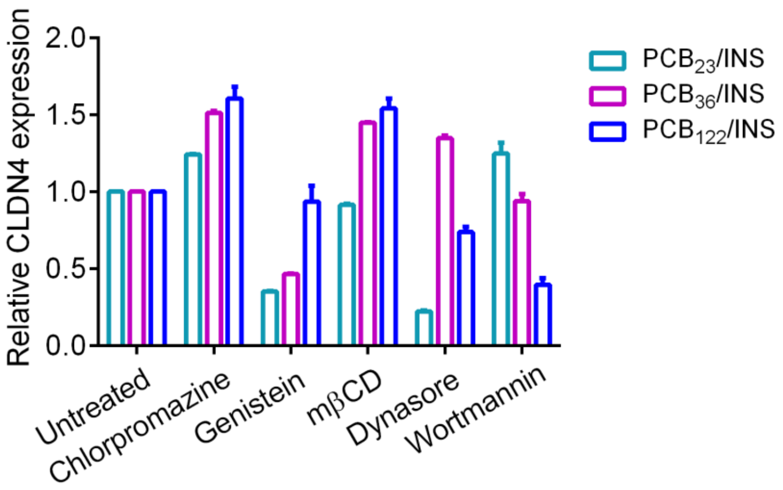

Figure S11. Quantified results of Western blot of CLDN4 protein in Figure 5B. The Western bands were scanned and normalized to the gray values of internal control β-actin. Western blot results in Figure 5B were quantified using ImageJ. The data was relative to the untreated control in each Western band. The untreated control was settled to 1. The mean ± SD was shown (n = 3).

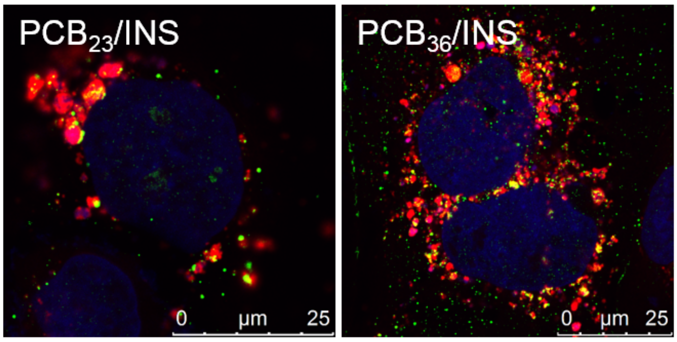

Figure S12. Fluorescence images of Caco-2 cell monolayers after incubation with PCB_{23}/INS and PCB_{36}/INS particles for 2 h. CLDN4 was shown in green. Endosomes/lysosomes stained with LysoTracker deep red were in red. Cell nuclei stained with DAPI were in blue.

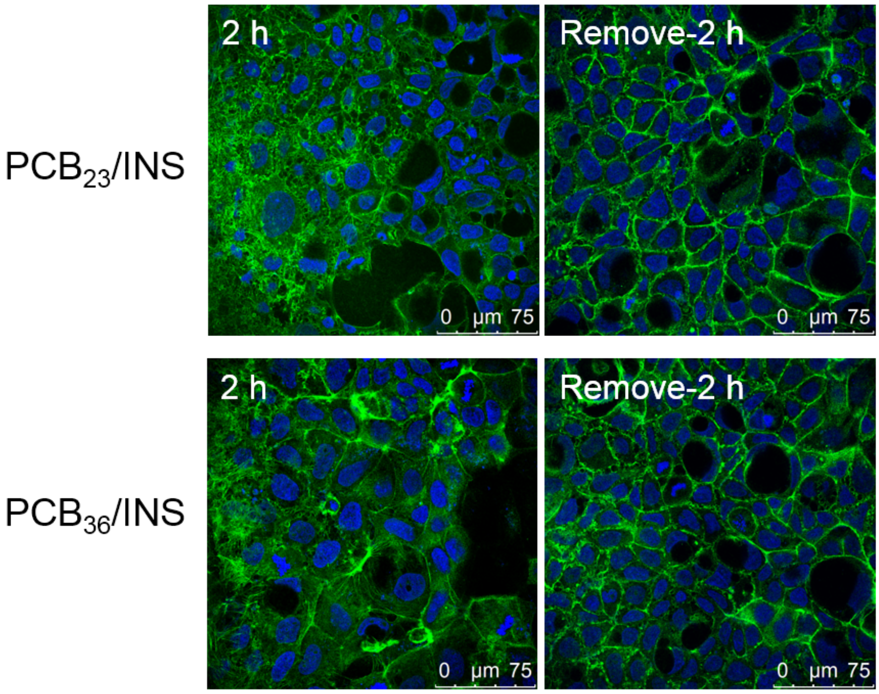

Figure S13. Fluorescence images of Caco-2 cell monolayers after incubation with PCB_{23}/INS and PCB_{36}/INS particles for 2 h and removing 2 h, respectively. Fluorescence images were collected by CLSM. F-actin stained with FITC-labeled phalloidin was shown in green, and the cell nuclei stained with DAPI were in blue.

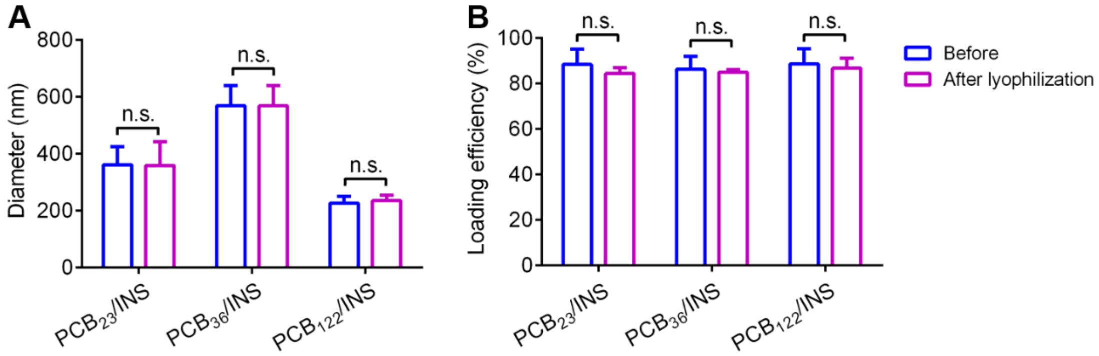

Figure S14. Characterization of PCB/INS particles after lyophilization. **(A)** Diameter detected by dynamic light scattering. **(B)** Loading efficiency of insulin. The mean ± SD was shown (n = 3). n.s. > 0.05.

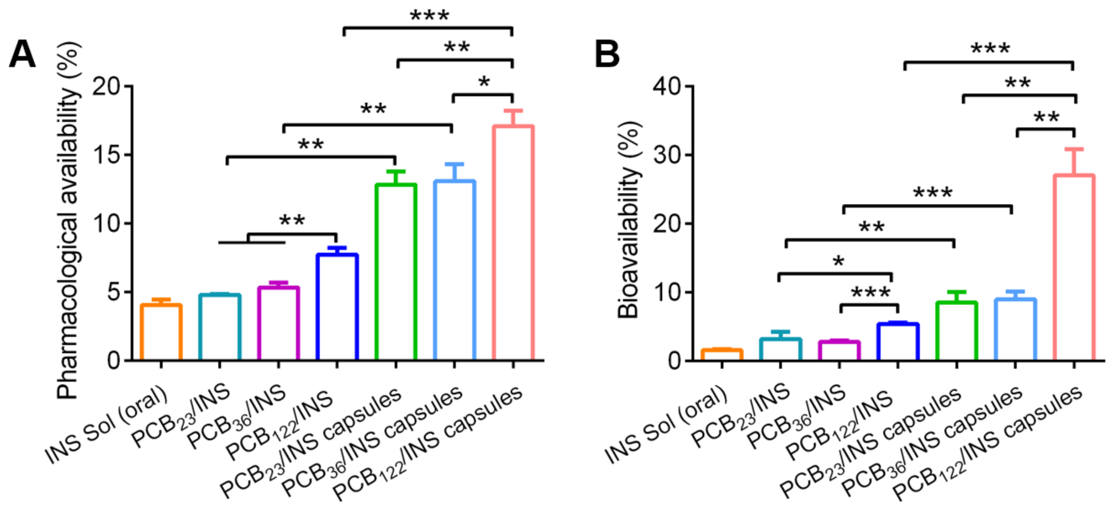

Figure S15. The pharmacological availability **(A)** and bioavailability **(B)** of insulin calculated from Figure 6A and 6B. The mean ± SD was shown (n = 5). *P < 0.05, **P < 0.01, ***P < 0.001.

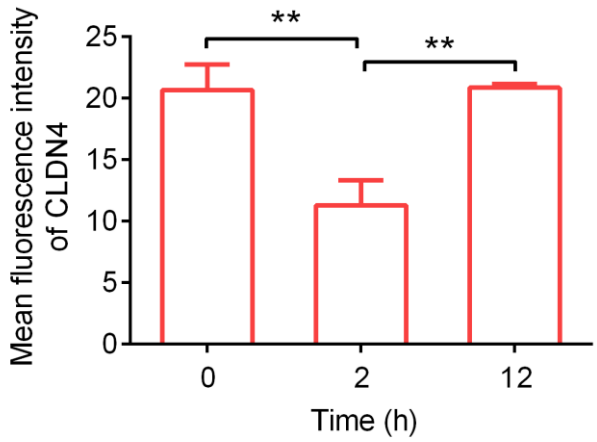

Figure S16. Quantified analysis of mean fluorescence intensity of CLDN4 protein in Figure 7B using ImageJ. The quantified analysis was normalized to the same area of tissues. The mean ± SD was shown (n = 3). **P < 0.01.
